# Screening, brief intervention, and referral to treatment training for Nigerian primary care physicians: A pilot evaluation of knowledge, attitudes, self-efficacy, and barriers to implementation

**DOI:** 10.1371/journal.pgph.0005597

**Published:** 2025-12-19

**Authors:** Honest Anaba, Johannes Thrul, Elohor Oborevwori, Osiyemi Oluwayomi, G. Caleb Alexander

**Affiliations:** 1 Department of Mental Health, Johns Hopkins Bloomberg School of Public Health, Baltimore, Maryland, United States of America; 2 Sidney Kimmel Comprehensive Cancer Center at Johns Hopkins, Baltimore, Maryland, United States of America; 3 Centre for Alcohol Policy Research, La Trobe University, Melbourne, Australia; 4 Department of Epidemiology, Johns Hopkins Bloomberg School of Public Health, Baltimore, Maryland, United States of America; 5 Lagos State Primary Healthcare, Eti-Osa LGA, Lagos, Nigeria; 6 Division of General Internal Medicine, Johns Hopkins Medicine, Baltimore, Maryland, United States of America; Health Policy Research Group, University of Nigeria, NIGERIA

## Abstract

Substance use disorder (SUD) poses a significant public health challenge in sub-Saharan Africa, where evidence-based approaches such as Screening, Brief Intervention, and Referral to Treatment (SBIRT) are rarely integrated into primary healthcare. In Nigeria, primary care providers often lack substance use prevention training, limiting their ability to identify and manage individuals with risky substance use patterns. This pilot study is the first documented evaluation of structured SBIRT training delivered to physicians within Nigeria’s primary healthcare system, assessing changes in self-reported knowledge, attitudes, delivery self-efficacy, and perceived implementation barriers. A single-group, pre–post pilot study was conducted with primary care physicians selected by the Lagos State Primary Healthcare Board. Of 33 enrolled, 25 (76%) completed both baseline and post-training assessments after a two-day train-the-trainer SBIRT course. Baseline data were summarized descriptively, and pre-post changes were analyzed using Wilcoxon signed-rank tests with rank-biserial effect sizes and 95% confidence intervals.At baseline, 9% (3/33) had prior SBIRT training, 79% (26/33) were unaware of validated screening tools, 36% (12/33) believed all SUD cases required referral regardless of severity, and 33% (11/33) viewed SUD as a moral failing. Post-training, significant changes were observed across all self-reported domains (r = 0.45–0.85, p < 0.05). Training satisfaction was high (94%), although time constraints and clinical workload were cited as key SBIRT implementation barriers. In this pilot study, structured SBIRT training was associated with improvements in primary care physicians’ self-reported knowledge, attitudes, and delivery self-efficacy. Addressing time and workload barriers may support sustainable implementation in the Nigerian primary healthcare system.

## Introduction

Substance use disorder (SUD) is a major global health concern, affecting an estimated 64 million individuals worldwide [1]. In sub-Saharan Africa, over 40% of alcohol consumers engage in heavy episodic drinking [2,3], while Nigeria has seen marked increases in non-medical opioid use, particularly tramadol and codeine among young adults [4]. The 2018 National Drug Use Survey estimated that nearly three million Nigerians met diagnostic criteria for SUD [4]. However, two-thirds of high-risk individuals needing treatment could not access appropriate services [4]. Key barriers include stigma, limited access, inadequate infrastructure, and insufficient provider training in evidence-based interventions [3–5].

In response to this growing burden, Nigeria’s National Drug Control Master Plan (NDCMP 2021–2025) outlines a strategy to integrate substance use prevention and treatment services into the primary healthcare (PHC) system [5]. PHC facilities accounts for 85.3% of the country’s healthcare infrastructure, and serve as the first point of contact for most patients [1,5,6]. The plan emphasizes a shift from specialist-led to generalist-led care, supporting early detection and intervention [5]. However, despite this policy intent, routine screening and brief intervention for substance use have remained uncommon in PHC, reflecting a persistent gap between strategic goals and clinical practice [4,5].

Screening, Brief Intervention, and Referral to Treatment (SBIRT) is a structured, evidence-based approach designed to identify and address risky substance use within healthcare settings [7^,^^8^]. In high-income countries, SBIRT reduces hazardous use by 10–30%, improve treatment engagement, and lower long-term healthcare costs, with sustained effects over 12–48 months [7,9,10]. Its low-intensity, adaptable format makes it particularly suitable for resource-constrained settings where specialist services are scarce [11,12].

Although SBIRT has been piloted in specialized settings in sub-Saharan Africa, including emergency departments, antenatal services, and HIV clinics, its integration into routine PHC remains limited [12–14]. In Nigeria, health extension workers and tertiary hospital physicians have delivered SBIRT interventions targeting tobacco and alcohol use in semi-rural areas [15–17]. These studies demonstrated feasibility and potential impact. However, they were focused on community outreach models or tertiary care settings, not facility-based PHC providers such as physicians and nurses, who are central to daily clinical care [5,12]. Moreover, these efforts excluded commonly used substances in Nigeria such as opioids, synthetic cannabis, and stimulants [15,17,18].

Given the reach of Nigeria’s PHC system, training frontline clinical providers in SBIRT represents a critical step toward achieving the early intervention goals outlined in national policy [5]. To address this gap, we piloted a two-day SBIRT training for lead PHC physicians, delivered through a train-the-trainer model, an approach that supports scalability [14]. To our knowledge, this appears to be the first documented evaluation of structured SBIRT training delivered to physicians within Nigeria’s public-sector PHC system. We assessed changes in self-reported knowledge, attitudes, and delivery self-efficacy, and explored perceived barriers to implementation. Findings aim to inform future efforts to integrate SBIRT into routine PHC practice in Nigeria and other resource-limited settings.

## Materials and methods

### Ethical consideration

This study was approved as exempt research by the Institutional Review Board (IRB) of the Johns Hopkins Bloomberg School of Public Health. All participants provided informed consent before enrollment. Additional information on the ethical, cultural, and scientific considerations specific to inclusivity in global research is included in the Supporting Information (S1 File). All materials and de-identified data are publicly accessible at https://osf.io/fn2qy/.

### Literature search

A focused literature search was conducted in PubMed, Embase, and Google Scholar in March 2025 using combinations of the terms: ‘Brief Intervention,’ ‘physician,’ ‘primary care,’ ‘training,’ and ‘Nigeria.’ The search yielded no prior published evaluations of SBIRT training among physicians in Nigeria’s public-sector primary healthcare system.

### Study design, and population

We employed a single–group, pre–post design to evaluate a structured SBIRT training for primary care physicians in Lagos State, Nigeria. This design generated preliminary evidence on feasibility and short-term changes in self-reported outcomes. Physicians were pre-selected by the Lagos State Primary Healthcare Board based on leadership roles within their respective local government areas and prior public health programs involvement. This sampling approach prioritized those positioned to cascade SBIRT knowledge to colleagues through a train-the-trainer model.

Eligible participants were physicians aged ≥18 years, currently practicing in public-sector PHC facilities across designated LGAs (Local Government Areas). Non-physicians and those practicing outside the selected LGAs were excluded. Of 33 physicians who completed the baseline survey, 25 (75.8%) completed the post-training assessment (S1 Fig). Follow-up reminders were sent via WhatsApp to enhance response completion.

### Intervention

The two-day, in-person SBIRT training was delivered by clinical and behavioral health experts and covered key content areas aligned with the study’s focus. Curriculum topics included: The epidemiology and neurobiology of substance use, validated screening tools such as Tobacco, Alcohol, Prescription medication, and other Substances (TAPS), Alcohol, Smoking and Substance Involvement Screening Test (ASSIST)-NIDA–modified, and others, motivational interviewing techniques including the Brief Negotiated Interview (BNI), and referral guidelines contextualized to local treatment systems. The training also addressed stigma and discrimination in addiction. Emphasis was placed on tools that screen multiple substances simultaneously, reflecting Nigeria’s poly-substance use trends (S5 File)

Instructional methods included video demonstrations, group discussions, interactive role-play, and question-and-answer sessions. All participants received 10 Continuing Medical Education (CME) credits from the Nigerian Medical Association upon completion.

The SBIRT training curriculum (S2 File), slide deck (S3 File), and pre–post survey instruments (S4 File) are provided in supplemental documents.

### Measures

We administered structured online surveys immediately before and after the two-day SBIRT training. Items assessed provider perceived knowledge, attitudes, delivery self-efficacy, training satisfaction, and perceived implementation feasibility. Primary outcomes were self-reported changes in perceived knowledge, attitudes, and delivery self-efficacy regarding SBIRT, analyzed as paired observations. Secondary outcomes included post-training satisfaction and qualitative responses on perceived implementation readiness and barriers. We collected all data electronically using Google Forms.

The questionnaire was developed using the Kirkpatrick training evaluation model to capture participants’ immediate reactions and learning outcomes [19]. Items were informed by established SBIRT evaluation frameworks particularly for knowledge, attitudes, and self‑efficacy, adapted to the local context [12]. Training facilitators and co-authors reviewed the draft to ensure content relevance, clarity, and cultural appropriateness.

To reduce respondent burden and promote survey completion, we used single item to assess participants’ self-rated knowledge, and attitudes for training objectives. While we did not conduct full psychometric validation due to pilot constraints, a multi-item self-efficacy scale demonstrated good internal consistency (Cronbach’s α = 0.81 (95% CI: 0.64–0.91) pre-training; 0.84 (95% CI: 0.71–0.92) post-training). Participants also reported perceived barriers to SBIRT implementation through open-ended questions. Responses were analyzed inductively and summarized into thematic categories.

### Statistical analysis

We used descriptive statistics to summarize baseline characteristics, training satisfaction, and perceived implementation feasibility following training. Differences in demographic characteristics between analytical sample and participants who dropped out were analyzed using Fisher’s exact test, given small cell sizes.

To evaluate participant changes in knowledge, attitudes, and delivery self-efficacy, we analyzed paired responses from only participants who completed both pre- and post-training surveys. The Shapiro–Wilk test was used to assess the normality of pre-post difference scores for ordinal outcomes. As assumptions for normality were not met, we used the Wilcoxon signed-rank test to compare pre-post changes. No imputation was performed for missing data. Given this is an exploratory pilot and hypothesis-generating aim, we did not adjust for multiplicity effect sizes were calculated using rank-biserial correlation, with 95% confidence intervals generated via bootstrapping (10,000 replicates). Effect sizes were interpreted according to Cohen’s guidelines: small (*r* ≈ 0.1), medium (*r* ≈ 0.3), and large (*r* ≥ 0.5). All analyses were conducted using R version 4.3.0.

## Results

### Participant characteristics

Among participants included in the analytic sample (n = 25), 56.0% were male, and the majority were between 35 and 44 years of age (52.0%). Most were married (72.0%), identified as Christian (80.0%), and reported five years or less of clinical experience (40.0%).

In contrast, non-completers (n = 8) were slightly more likely to be female (62.5%), younger (25.0% were aged 18–24 and 50.0% were 25–34), and single (62.5%). No statistically significant differences in baseline demographics were observed between completers and non-completers (all *p* > 0.05, Fisher’s exact test) (Table 1).

**Table 1:** Participant demographics and comparison between completers and non–completers.

Characteristics	Pre-SBIRT n = 33 (%)	Post-SBIRT (completers) n = 25 (%)	Noncompleters n = 8 (%)	*p*-value^a^
Sex Male Female	17 (51.5) 16 (48.5)	14 (56.0) 11 (44.0)	5 (62.5) 3 (37.5)	0.44
Age, years 18 - 24 25 - 34 35 - 44 45 - 54	2 (6.0) 12 (36.4) 14 (42.4) 5 (15.2)	0 (0) 8 (32.0) 13 (52.0) 4 (16.0)	2 (25.0) 4 (50.0) 1 (12.5) 1 (12.5)	0.05
Marital Status Single Married	12 (36.4) 21 (63.6)	7 (28.0) 18 (72.0)	5 (62.5) 3 (37.5)	0.11
Religion Christian Muslim	26 (78.8) 7 (21.2)	20 (80.0) 5 (20.0)	6 (75.5) 2 (24.5)	1.00
Clinical Experience, years Five or less Six to ten Eleven or more	16 (48.4) 11 (33.3) 6 (18.2)	10 (40.0) 10 (40.0) 5 (20.0)	6 (75.0) 2 (12.5) 1 (12.5)	0.12

^a^p-values calculated using Fisher’s exact test comparing completers and non-completers

### Baseline knowledge and attitudes and perception

At baseline, only 9.1% of participants reported prior formal SBIRT training. Only 21.2% were familiar with validated screening tools for multiple substances, and 18.2% reported previous exposure to the SBIRT model. Two-thirds (66.7%) reported confidence in recognizing SUD symptoms, while 33.3% reported regular clinical encounters with patients presenting with substance use issues.

All respondents (100%) agreed that primary care providers play a critical role in addressing SUD. However, 36.4% believed all SUD cases should be referred to specialists regardless of severity. Only 60.7% of participants agreed or strongly agreed that “SUD is a medical issue, not a moral failing”, while 21.2% were neutral and 18.2% disagreed or strongly disagreed (Table 2).

**Table 2:** Baseline knowledge, attitudes towards SUD and SBIRT.

Measures	Pre-SBIRT N = 33 (%)
Knowledge	
Prior SBIRT Training Yes No	3 (9.1) 30 (90.9)
Frequency of encounter with Known SUD patient Never/Rarely Sometimes Often/Always	7 (21.2) 15 (45.5) 11 (33.3)
Aware of validated tools to screen tobacco, alcohol and other drugs Yes No	7 (21.2) 26 (79.8)
Aware of SUD signs and symptoms Yes No	22 (66.7) 11 (33.3)
Aware of SBIRT as an assessment approach Yes No Not sure	6 (18.2) 21 (63.6) 6 (18.2)
Attitudes	
Primary care providers play key role in assessing substance use Yes No	33 (100) 0 (0)
All SUD cases should be referred regardless of severity Yes No Not Sure	12 (36.4) 17 (51.5) 4 (12.1)
SUD is a medical issue, not moral failing Agree/Strongly Agree Neutral Disagree/ Strongly Disagree	20 (60.7) 7 (21.2) 6 (18.2)

This table summarizes baseline SBIRT knowledge and attitudes prior to training among 33 participating physicians.

### Pre–post changes in knowledge, attitudes and self-efficacy

Post-training, the proportion rating their assessment knowledge as excellent increased from 0% to 68%, and those very confident in using validated screening tools increased from 4% to 68%. The proportion very likely to treat patients with SUD as they would other chronic conditions increased from 32% to 68% (Table 3).

**Table 3:** Descriptive pre-post changes in knowledge, attitudes, and self-efficacy.

Measures	Response Category	Pre-SBIRT n = 25 (%)	Post-SBIRT n = 25 (%)
Knowledge			
Assessing Substance use	Excellent Good Average Poor	0 (0) 3 (12) 14 (56) 8 (32)	17 (68) 8 (32) 0 (0) 0 (0)
Attitudes			
Likelihood treating SUD patients with respect as others	Very likely Likely Somewhat likely Not likely	8 (32) 12 (48) 4 (16) 1 (4)	17 (68) 7 (28) 1 (4) 0 (0)
Self-Efficacy			
Confidence starting conversations	Very Confident Confident Somewhat Confident Not Confident	9 (36) 8 (32) 7 (28) 1 (4)	11 (44) 13 (52) 1 (4) 0 (0)
Confidence screening with validated instruments	Very Confident Confident Somewhat Confident Not Confident	1 (4) 3 (12) 6 (24) 15 (60)	17 (68) 8 (32) 0 (0) 0 (0)
Confidence in brief Intervention	Very Confident Confident Somewhat Confident Not Confident	3 (12) 7 (28) 10 (40) 5 (20)	15 (60) 8 (36) 1 (4) 0 (0)
Confidence initiating appropriate referrals	Very Confident Confident Somewhat Confident Not Confident	5 (20) 13 (52) 5 (20) 2 (8)	15 (60) 10 (40) 0 (0) 0 (0)

This table presents the distribution of pre–post responses for SBIRT knowledge, attitudes, and delivery self-efficacy among 25 participants completing both assessments.

All changes were statistically significant (Table 4). Median self-reported knowledge scores improved from 2 (IQR: 1–2) to 4 (3–4) (p < 0.001, *r* = 0.80). Attitude item improved from 3 (IQR: 2–4) to 4 (IQR: 3–4) (p = 0.006, *r* = 0.55). Significant gains were observed in delivery self-efficacy for initiating conversations from 3 (IQR: 2–4) to 3 (IQR: 3–4) (*p* = 0.017, *r* = 0.45), screening from 1 (IQR: 1–2) to 4 (IQR: 3–4) (*p* < 0.001, *r* = 0.86), delivering brief interventions from 2 (IQR: 2–3) to 4 (IQR: 3–4) (*p* < 0.001, *r* = 0.79), and making referrals from 3 (IQR: 2–3) to 4 (IQR: 3–4) (*p* = 0.002, *r* = 0.72).

**Table 4:** Statistical comparison of pre-post changes in knowledge, attitude, and delivery self-efficacy.

Measures	Pre-SBIRT Median (IQR)	Post-SBIRT Median (IQR)	Test Statistic(Z)	*p*-value	Effect size(*r*)	95% CI
Knowledge						
Assessing Substance use	2 (1–2)	4 (3–4)	4.28	<0.001	0.80	[0.75–0.90]
Attitudes						
Treating SUD patients like others	3 (2–4)	4 (3–4)	2.72	0.006	0.55	[0.29–0.71]
Delivery Self- Efficacy						
Confidence starting talks	3 (2–4)	3 (3–4)	2.31	0.017	0.45	[0.11–0.69]
Confidence screening	1 (1–2)	4 (3–4)	4.37	<0.001	0.86	[0.85–0.91]
Confidence Brief Intervention	2 (2–3)	4 (3–4)	3.77	<0.001	0.79	[0.62–0.88]
Confidence Referring	3 (2–3)	4 (3–4)	3.01	0.002	0.72	[0.47– 0.87]

Median scores are based on a 4-point Likert scale. p-values from Wilcoxon signed-rank test. Effect sizes are rank-biserial correlations.

### Training satisfaction and implementation readiness

Most participants reported high levels of satisfaction with the training. A majority rated the content as very clear and practical (76%), and 96% reported being likely to implement SBIRT in their clinical practice. No participants reported being unlikely to apply the training (Table 5).

**Table 5:** Post-training feedback & implementation intentions.

Measures	Response Distribution n = 25 (%)
Overall Satisfaction with training	Very Satisfied 17 (68), Satisfied 7 (28), Very Dissatisfied 1 (4)
Clarity & Practicality of Materials	Very Good 19 (76), Good 5 (20), Average 1 (4)
Interactivity & Engagement	Very good 19 (76), Good 6 (24)
Satisfaction with training duration	Very Satisfied 6 (24), Satisfied 12 (48), Neutral 7 (28)
Likelihood of Implementing SBIRT within three months	Very Likely 12 (48), likely 12 (48), Somewhat likely 1 (4)

This table summarizes participant satisfaction and intention to implement SBIRT following the training.

### Changes in reported barriers to SBIRT implementation

Post-training, the most frequently reported barriers were time constraints or clinical workload (52%). Patient resistance was cited by 16%, systemic or referral issues by 4%, cultural or language concerns by 4%, and other barriers by 8%.

Before the intervention, 80% cited lack of training as a barrier before the intervention; this was not reported afterward. Mentions of time constraints (28%), patient resistance (32%), and systemic barriers (24%) also declined post-training (Table 6).

**Table 6:** Comparison of pre–post changes in reported barriers to SBIRT implementation.

Barrier Themes	Pre training n = 25(%)	Post training n = 25(%)
Lack of training	20 (80.0)	–
Time Constraints/ Clinical workload	7 (28.0)	13 (52.0)
Patient resistance/ readiness	8 (32.0)	4 (16.0)
Systemic issues/ Referral barriers	6 (24.0)	1 (4.0)
Cultural/language issues	–	1 (4.0)
Other	1 (4.0)	2 (8.0)

Participants could report multiple barriers. Themes were inductively coded from open-ended responses. (–) means not mentioned or not applicable.

## Discussion

This study addresses a critical gap in SBIRT implementation research from sub-Saharan Africa by evaluating outcomes among facility-based PHC physicians in Nigeria, a group central to routine care but often excluded from previous SBIRT efforts [5,12]. Unlike earlier south-western Nigerian studies involving community health extension workers (CHEWs) in semi-rural outreach [15,17,20], this pilot assessed SBIRT training within standard PHC clinics. In this context, brief, structured training was associated with changes in self-reported provider knowledge, attitudes, and delivery self-efficacy related to risky substance use, and was perceived as both feasible and acceptable. These findings offer timely implementation insights and align with Nigeria’s NDCMP (2021–2025), contributing foundational evidence for integrating substance use prevention into PHC in low-resource settings [3–5,11].

Measurable changes were observed across three domains in this study. First, participants gained awareness on how to identify and respond to risky substance use, addressing gaps in knowledge [12,21]. Second, participants expressed more respectful attitudes toward individuals with SUD, as reflected in a greater likelihood of treating them like patients with other chronic conditions. While stigma was not formally assessed, this shift may suggest an increased recognition of SUD as a chronic health condition deserving equitable care [22–25]. Third, self-efficacy improved across the SBIRT components—initiating conversations, screening, brief intervention, and referral, indicating enhanced clinical readiness to engage patients and support earlier intervention [17]. These findings are consistent with the possibility that even brief, structured training may shift provider perspectives and build core competencies for engaging at-risk individuals in primary care [10,26,27].

The training was also perceived as relevant, feasible, and applicable. Partcipants reported strong intentions to integrate SBIRT into their daily practice, reinforcing its suitability for incorporation into routine PHC delivery, clinical education, and continuing professional development [24,26,28–30]. One observed change in perceived feasibility was the disappearance of “lack of training” as a reported barrier post-intervention, which may indicate that participants no longer viewed inadequate preparation as an obstacle to SBIRT delivery [31]. Similar reductions were observed in reported patient resistance and referral barriers, which may reflect increased awareness of communication strategies and available service pathways introduced during sessions. Collectively, these changes are consistent with potential improvement in provider readiness to navigate common implementation challenges [10,31,32]. 

Still, structural barriers, including time constraints and workload, were frequently cited by most participants, a finding widely reported across Africa brief intervention studies [13,18,20]. Anticipating these challenges, the training incorporated the Brief Negotiated Interview (BNI), a semi-structured, evidence-based technique designed for busy healthcare settings [30,31]. BNI has been shown to enhance treatment engagement without requiring lengthy consultations or advanced motivational interviewing skills [31]. However, as shown in the South African Teachable Moment study [13], sustainable change requires more than time-efficient provider training. It depends on workflow alignment, leadership support, and organizational readiness [9,18,20,32].

This study addresses several key gaps in the regional evidence base. In Nigeria, prior SBIRT efforts largely focused on alcohol and tobacco use [15^,^^17^]. In contrast, our study broadened the scope to include opioids, stimulants, synthetic cannabinoids, and other substances, reflecting the country’s shifting poly-substance use trends [1,4,5]. It also complements broader regional initiatives, such as South Africa’s SBIRT-in-HIV-care model, which employed a train-the-trainer cascade [14]. While our study did not assess fidelity or system-level integration, these regional models provide practical insights for future scale-up, particularly around hybrid training formats, supportive supervision, and health system alignment [33,34].

Building on this train-the-trainer approach, the next phase will assess key implementation outcomes such as feasibility, fidelity, and penetration, to evaluate scalability across Nigeria’s primary healthcare system. Physicians from the pilot will mentor and train colleagues within local primary healthcare centers. These sessions create successive waves of learning, extending SBIRT skills from a small core group to nurses and community health workers. Each wave emphasizes hands-on practice, peer support, and feedback to maintain consistency. Over time, this cascading model aims to embed SBIRT into everyday clinical routines and strengthen Nigeria’s primary care response to substance use.

Nevertheless, West Africa remains underrepresented in SBIRT studies [13]. A recent scoping review highlighted this imbalance, noting the predominance of studies from Southern and Eastern Africa, as well as a lack of rigorous evaluations of provider training outcomes [13], a gap this pilot study addresses. Beyond its contributions to regional gaps, this study has several strengths. It evaluates an underexplored group (PHC physicians) using a train-the-trainer approach suited for scaling. Evidence suggests that SBIRT implementation in PHC is more cost-effective than in specialist settings, supporting the relevance of this focus [33]. It also incorporates practical, time-efficient tools, such as BNI, and uses appropriate non-parametric tests and effect size reporting to quantify changes and ensure analytical rigor, despite the small sample size.

However, this study has several limitations that warrant careful interpretation. First, the single-group pre-post design cannot rule out alternative explanations for observed changes, Including testing effects and social desirability bias. Without a control group, we cannot establish the training, rather than other concurrent factors caused the observed changes. All findings should be considered exploratory rather than confirmatory.

Second, outcomes relied exclusively on self-reported, single-item measures administered immediately after the training. The measures were not formally validated in the Nigerian context, and single-item assessments, while practical, offer less reliability than multi-item validated scales. The immediate post-training assessment may inflate effect sizes due to recency and social desirability effects.

Third, the small convenience sample (n = 25 complete cases) from one state limits statistical power and generalizability to Nigeria’s broader PHC workforce. The purposive selection of physician leaders may introduce selection bias, as these individuals may be more motivated or capable than typical PHC providers.

Fourth, implementation barriers were assessed through open-ended responses rather than structured, validated implementation science frameworks (e.g., Consolidated Framework for Implementation Research [CFIR], Theoretical Domains Framework [TDF]), limiting systematic comparison with other studies. Finally, we did not assess training fidelity, measure actual clinical practice changes or include patient outcomes, all of which are necessary to determine real-world implementation success.

## Conclusion

This pilot study provides preliminary evidence that brief, structured SBIRT training is associated with improvements in Nigerian PHC physicians’ self-reported knowledge, attitudes, and delivery self-efficacy for addressing SUD, along with high perceived feasibility and acceptability. However, training alone is insufficient for sustainable implementation. Successful integration will require addressing structural barriers including time constraints, workload pressures, and limited referral pathways through system-level support, organizational readiness and alignment with existing clinical workflows . Future research should evaluate the cascading train-the-trainer model’s fidelity, assess actual clinical practice changes, and measure patient-level outcomes to determine real world effectiveness to inform national scale-up efforts.

## Supporting information

S1 File Inclusivity in global research questionnaire. (PDF)

S2 File SBIRT training curriculum. (PDF)

S3 File Slide deck. (PDF)

S4 File Pre-post survery instruments. (DOCX)

S1 Fig SBIRT participant CONSORT. (PDF)

S5 File TiDier checklist SBIRT. (PDF)
