# Supplementary material for: Screening, brief intervention, and referral to treatment training for Nigerian primary care physicians: A pilot evaluation of knowledge, attitudes, self-efficacy, and barriers to implementation
Source: PLOS Glob Public Health. 2025 Dec 19;5(12):e0005597. doi: 10.1371/journal.pgph.0005597 (PMC12716713; doi:10.1371/journal.pgph.0005597)
Supplement: S1 File — (PDF) [file pgph.0005597.s001.pdf]

Inclusivity in global research

PLOS' policy on inclusivity in global research aims to improve transparency in the reporting of research performed outside of researchers' own country or community and ensures that PLOS publications reporting global research adhere to high standards for research ethics and authorship. Authors of relevant research articles may be asked to complete the questionnaire below, which outlines ethical, cultural, and scientific considerations specific to inclusivity in global research. This questionnaire may be requested when researchers have travelled to a different country to conduct research, if research uses samples collected in another country, research with Indigenous populations or their lands, or if research is on cultural artefacts. Researchers travelling to another country solely to use laboratory equipment will not normally be required to complete the questionnaire. However, the questionnaire can be requested at the journal's discretion for any submission – if you have been requested to complete this questionnaire by the PLOS journal you submitted to, please do so.

Please complete the questionnaire below and include this as a Supporting Information file with your manuscript. Note that if your paper is accepted for publication, this checklist will be published with your article in the supporting information files. Please ensure that you reference the checklist in the main body of your manuscript. We suggest adding a subsection 'Inclusivity in global research' to your Methods section and adding the following sentence: "Additional information regarding the ethical, cultural, and scientific considerations specific to inclusivity in global research is included in the Supporting Information (SX Checklist)"

The questions have been designed to be applicable to a wide range of study types, and there are subsections for both human subjects research and non-human subjects research. If any of the questions are not relevant to your research please mark them as "N/A" as appropriate.

Ethical considerations, permits and authorship

This section is applicable to all research types.

Provide details as to who granted permissions and/or consent for the study to take place in the Methods section of your manuscript. This should include the names of **all** ethics boards, governmental organizations, community leaders or other bodies that provided approval for the study. If individuals provided approval refer to these people by their role or title but do not list their name(s).

The study was reviewed by the Johns Hopkins Bloomberg School of Public Health Institutional Review Board (IRB) and determined to be 'Not Engaged in Human Subjects Research' under U.S. DHHS regulations (45 CFR 46.102), as the evaluation assessed a quality improvement training program using de-identified survey data. All participants provided written informed consent prior to participation. No minors were included."

If there were any deviations from the study protocol after approval was obtained please provide details of

Reported on page number:
No deviations from the study protocol occurred after approval was obtained.

these changes in the Methods section of your manuscript.
Did this study involve local collaborators that are residents of the country where the research was conducted or members of the community studied? If you do not have any authors from said communities, please provide an explanation for this below.

Yes. This study involved substantive collaboration with Nigerian colleagues who are co-authors on this manuscript:

- Drs Honest Anaba and Elohor Oborevwori (co-authors): Nigerian Physicians affiliated with Johns Hopkins Bloomberg School of Public Health, provided executed project collaborated with key stakeholders in the cultural adaptation of training content.
- Osiyemi Oluwayomi (co-author): Physician affiliated with Lagos State Primary Healthcare, Eti-Osa LGA, Lagos, Nigeria. Dr. Osiyemi provided critical input on PHC operational realities, facilitated recruitment through the Lagos State PHC Board, and co-delivered training modules.

Additionally, the training facilitation team included Nigerian experts in clinical psychology, mental health, and addiction services who co-designed and co-delivered the intervention. Guest speakers from Nigerian governmental organizations (National Drug Law Enforcement Agency, National Agency for Food and Drug Administration and Control) and professional associations (Nigerian Medical Association, International Society of Substance Use Professionals Nigeria chapter) provided contextual expertise and policy perspectives

Everyone listed as an author should meet PLOS' criteria for authorship and all individuals who meet these criteria should be included in the author byline, rather than the acknowledgements. For further information please see the journal's Authorship Policy.

Human subjects research (e.g. health research, medical research, cross-cultural psychology)

Did you obtain written informed consent from a representative of the local community or region before the research took place? How did you establish who speaks for the community? Details of written informed consent obtained from study participants should be reported separately in the Methods section of your

Administrative approval was obtained to conduct training and administer surveys from the Dr Akinolu, Lagos State Primary Healthcare Board, the governmental body responsible for PHC oversight in Lagos State. The Board identified and pre-selected 'lead physicians' from Local Government Areas (LGAs) across Lagos State based on their leadership roles and involvement in public health programs. Individual participants provided written informed consent prior to completing baseline surveys and participating in the

manuscript.

How did members of the local community provide input on the aims of the research investigation, its methodology, and its anticipated outcome(s)?

Pre-training (design phase):

Nigerian co-authors (EO, OO) reviewed study objectives, survey instruments, and training curriculum for cultural appropriateness, contextual relevance, and alignment with Nigeria's National Drug Control Master Plan (2021-2025).

Training content was adapted to reflect Nigerian substance use epidemiology (e.g., alcoholic beverages, cannabis, tramadol, codeine trends), local referral pathways, and Lagos-specific treatment resources.

During training:

Guest speakers from Nigerian agencies (NDLEA, NAFDAC, NMA) provided policy context and practical guidance on integrating SBIRT into Nigerian PHC workflows.
Participants provided real-time feedback on training content relevance and implementation feasibility through daily evaluations.

Post-training:

Close and Open-ended survey questions solicited participants' perspectives on anticipated implementation barriers and needed supports, which informed the study's recommendations for future scale-up."

When engaging with the local community, how did you ensure that the informed consent documents and other materials could be understood by local stakeholders?

All study materials (informed consent documents, survey instruments, training materials) were developed in English, the official language of instruction in Nigerian medical education and the working language of Nigerian PHC facilities. To ensure comprehension:

1. Consent documents: Reviewed by Nigerian co-authors and training facilitators for clarity and appropriateness. Written at a level suitable for physicians (medical professionals with university education).
2. Training materials: Reviewed by Nigerian co-authors and Addiction professionals. Materials included culturally relevant examples, local substance use statistics, and Lagos-specific referral resources.
3. In-person administration: The lead author (HA) and Nigerian co-facilitators were present during consent and survey administration to answer questions and clarify any unclear items. Participants had oportunities to ask questions before signing consent forms."

Will the findings of the research be made available in an understandable format to stakeholders in the community where the study was conducted (e.g. via a presentation, summary report, copies of publications, etc.)? Please provide details of how this will be achieved.

Yes. We are committed to disseminating findings to local stakeholders through multiple channels:

1. Direct dissemination to participants: Plain-language summary report (2-3 pages) will be shared with all participating physicians and the Lagos State Primary Healthcare Board within 30 days of manuscript acceptance. Summary will highlight key findings (training effectiveness, implementation barriers, recommendations) in accessible language without statistical jargon.

2. Presentation to Lagos State PHC leadership: Results presentation to Lagos State Primary Healthcare Board leadership to inform future SBIRT scale-up decisions and workforce development planning.

3. Policy brief: One-page policy brief for Nigerian governmental agencies (Federal Ministry of Health, National Drug Law Enforcement Agency) and professional associations (Nigerian Medical Association, ISSUP Nigeria) summarizing implications for national substance use policy implementation.

4. Open-access publication: This manuscript will be published in PLOS Global Public Health, an open-access journal, ensuring Nigerian researchers, policymakers, and practitioners can access the full findings without subscription barriers.

5. Public data repository: All de-identified data, training materials, and survey instruments are publicly available via Open Science Framework (OSF), enabling Nigerian researchers to conduct secondary analyses and adapt materials for local use.

Non-human subjects research using specimens/ animals collected as part of the study, or those housed in archival collections. Examples include archaeology, paleontology, botany and zoology.

Did the permission you obtained from a local authority to perform the study include an agreement on access to outputs and benefit sharing? This may include procedures to enable fair distribution of the benefits and resources arising from the research performed. Please include any details of Prior Informed Consent and Benefit Sharing Agreements obtained. These may be required by field-specific regulations, for example the Convention on Biological Diversity (CBD) and the associated Nagoya Protocol.

N/A

If the material used in your study was imported, please A) provide the year it was imported and B) indicate whether permits were obtained to import/export the materials used, C) provide details of any permits obtained. If this information is not available, please indicate this.

N/A

If you used archival specimens, please state how the material used in your study was acquired by the institute it is held in and provide details of any permits obtained for the original excavations/ sample collection. If this information is not available, please indicate this.

N/A

How was the potential cultural significance of the materials collected in your study to local communities considered in your research design? Were Indigenous peoples and/or local researchers and institutions involved with archaeological excavations / collection of specimens? If so, please provide a description of their involvement.

N/A

If your manuscript includes photographs of human remains please indicate whether authors obtained permission from descendants or affiliated cultural communities to do so.

N/A
