# Supplementary material for: Screening, brief intervention, and referral to treatment training for Nigerian primary care physicians: A pilot evaluation of knowledge, attitudes, self-efficacy, and barriers to implementation
Source: PLOS Glob Public Health. 2025 Dec 19;5(12):e0005597. doi: 10.1371/journal.pgph.0005597 (PMC12716713; doi:10.1371/journal.pgph.0005597)
Supplement: S2 File — (PDF) [file pgph.0005597.s002.pdf]

Johns Hopkins
Bloomberg School of Public Health

# SBIRT TRAINING CURRICULUM

Lagos State
Primary Health Care Board

# MEET THE TEAM

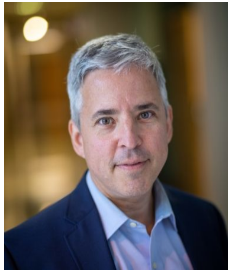

Caleb Alexander, MD, FACP
Professor of Epidemiology & Medicine,
School of Medicine & Public Health,
Johns Hopkins.
Project Preceptor

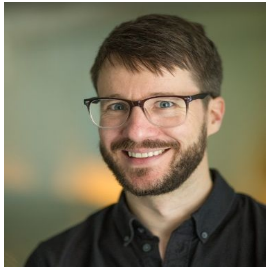

Johannes Thrul, PhD
Associate Professor,
Dept of Mental Health,
Bloomberg School of Public Health,
Johns Hopkins.
Project Preceptor

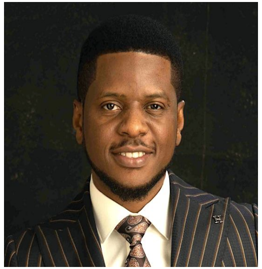

Honest Anaba, MBBS, FASAM
MPH Candidate,
Bloomberg School of Public Health,
Johns Hopkins.
Project Team Lead

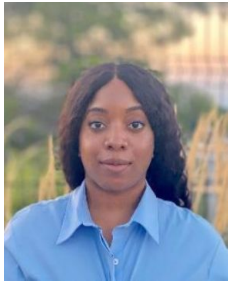

Ijebalim Nnachetta
MPH Candidate,
Bloomberg School of Public Health,
Johns Hopkins.
Project Team member

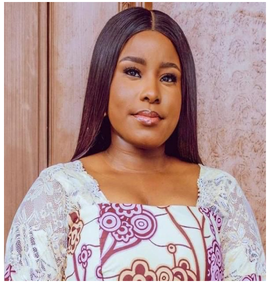

Elohor Oborevwori, MBBS
MPH Candidate,
Bloomberg School of Public Health,
Johns Hopkins.
Project Team member

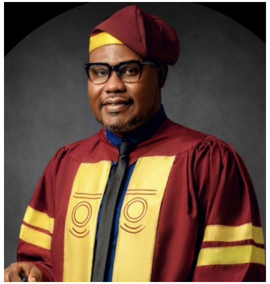

Osiyemi Oluwayomi, MBBS, MPH
Chief Medical Superintendent,
Eti-Osa, Primary Healthcare,
Lagos State.
Project Preceptor.

# OUTLINE

- Introduction to SBIRT
- Patterns, trends and scope of substance use in Nigeria
- Understanding Addiction
- Screening Overview
- Motivational Interviewing
- Brief Intervention Techniques
- Referral to treatment
- Destigmatizing Language in care settings

# OBJECTIVES

Understand the public health impact and progression of substance use disorders.

Learn to apply validated screening tools to identify at-risk individuals.

Develop skills in Motivational Interviewing (MI) and Brief Negotiated Interviews (BNI).

Master the referral process to connect patients with appropriate treatment and resources.

Foster a nonjudgmental, empathetic approach to patient interactions.

Integrate SBIRT practices into clinical workflows and collaborate with interdisciplinary teams.

# INTRODUCTION

Screening, Brief Intervention, and Referral to Treatment (SBIRT) is a comprehensive, integrated, public health approach to the delivery of early intervention and treatment services. It is used for:

- Persons whose use are at higher levels of risk.
- Persons who may already have a substance use disorder.
- Screening is useful for everyone.
- Brief intervention has been shown to be effective for unhealthy substance use, particularly alcohol use.

# SBIRT: A Transformative Approach to Addressing Substance Use

## Core components

SBIRT integrates Screening, Brief Intervention, and, when necessary, Referral to Treatment to address substance use effectively

## Traditional approaches

focused on universal prevention or specialized treatment for individuals meeting substance use disorder criteria, leaving gaps for at-risk populations

## Strategic Application

SBIRT offers a structured framework for managing medical and mental health issues linked to unhealthy alcohol or substance use

## Prevalence in Medical Settings

High rates of substance use are observed across primary care, hospitals, and emergency departments, regardless of whether the presenting complaint is substance-related.

## SBIRT bridges this gap

by adopting a public health perspective, targeting those at moderate or high risk for substance-related psychosocial or healthcare challenges

# Evidence of SBIRT Effectiveness in Primary Care

**Study Overview:** Ten diverse primary care practices implemented SBIRT for alcohol, drug use, depression, anxiety, and tobacco.

**Key Results**: Of 49,964 eligible patients, 36,394 were pre-screened, and 21,635 underwent full screening.

**Conditions Addressed:** Alcohol, drug use, depression, anxiety, tobacco, and child safety

**Impact**: Early detection enabled timely interventions, reducing risks before escalation.

**Best Practices for Success**: Practice champion, Interprofessional teams, Integration into workflows and EHRs, Ongoing training and strong referral networks.

Hargraves D, White C, Frederick R, et al. Implementing SBIRT (Screening, Brief Intervention and Referral to Treatment) in primary care: lessons learned from a multi-practice evaluation portfolio. *Public Health Reviews*. 2017;38(1). doi:10.1186/s40985-017-0077-0

# Screening Brief Intervention Referral to Treatment

## Truths

- Treatment does work
- The ED/Primary care visit is an opportunity for intervention
- Timely referral is effective
- Practitioners are reluctant to screen and intervene
- There are multiple barriers to the SBI

## Why Should we care?

- Prevalence
- Morbidity & Mortality
- Harm to Self & Others
- Economic Burden

# PREVALENCE, MORBIDITY & MORTALITY

1 in 7 persons (aged 15–64 years) had used a drug in the past year- NDUS.

1 in 5 individuals who had used drug in the past year is suffering from drug-related disorders.

Nigeria ranks 1st in Sub-Saharan Africa and 5th globally for deaths related to substance use disorders

Past-year drug use in the South-West zone is nearly double the national average, driven by high rates in Lagos and Oyo States.

European Union, United Nations Office on Drugs and Crime, Adewole IF, Kale Y. *Drug Use in Nigeria*.; 2018.https://www.unodc.org/documents/data-and-analysis/statistics/Drugs/Drug_Use_Survey_Nigeria_2019_Exsum.pdf
DRUG USE DEATH RATE BY COUNTRY. World Life Expectancy.https://www.worldlifeexpectancy.com/cause-of-death/drug-use/by-country/

# Key Patterns and Trends of Drug Use in Nigeria

**High Prevalence of Drug Use Among Men:**
Men are significantly more likely than women to use drugs, particularly cannabis. However, the gender gap is narrower for pharmaceutical opioids like tramadol and codeine.

**Mortality of Alcohol Use:**
Nigeria has one of the highest adult alcohol use rates in sub-Saharan Africa (40–60%), with NCDs accounting for 29% of all deaths

GBD 2016 Alcohol Collaborators Alcohol use and burden for 195 countries and territories, 1990–2016: a systematic analysis for the Global Burden of Disease Study 2016. Lancet. 2018;392:1015–1035. doi: 10.1016/S0140-6736(18)31310-2.

# ECONOMIC BURDEN OF SUBSTANCE USE

Smoked tobacco incurred 7 trillion naira in health expenditure, and smokeless tobacco 561.75 billion naira, representing 0.28% of the country's GDP between 2013–2020

Cannabis users spend an average of 2% of Nigeria’s minimum monthly wage on daily consumption, while cocaine users spend nearly 50%, highlighting the significant financial strain of substance use.

Adeniji, F. I. (2023). Economic burden of tobacco consumption in Nigeria, 2013–2020: A prevalence-based attributable-risk approach. *Population Medicine*, *5*(September), 1–10.https://doi.org/10.18332/popmed/171806

# Key Patterns and Trends of Drug Use in Nigeria

**Youth as the Most Affected Group:** Drug use is prevalent among individuals aged 25–39, particularly for cannabis, pharmaceutical opioids, and amphetamines. More concerns for public health is the dramatic rise in synthetic cannabis use(Colorado).

A study using the augmented CAGE questionnaire found harmful alcohol use among Nigerian undergraduates, with males at higher risk and alcohol-risk perception significantly predicting abuse

Vol. 16 No. 1 (2017) | African Journal of Drug and Alcohol Studies. https://www.ajol.info/index.php/ajdas/issue/view/16874

# National Drug Control Master Plan 2021-2025

- Integrate substance use treatment into primary healthcare (PHC) services across Nigeria by training PHC workers and CSOs in substance use and mental health management, enhancing infrastructure to enable services such as screening, brief intervention, treatment, aftercare, referral, and follow-up.

- This initiative aims to establish substance use treatment in PHCs in **at least two states per geopolitical zone**.

Inter-Ministerial Committee, Malami A, National Drug Law Enforcement Agency.*National Drug Control Master Plan 2021–2025*.https://www.unodc.org/conig/uploads/documents/NDCMP_2021-2025.pdf

## RATIONALE

- ➔ Screening and referral increases treatment contact.
- ➔ Monies saved.
- ➔ Improved prognosis.
- ➔ Medical Opportunity is a ‘teachable moment’

# Understanding Addiction

## Substance Use Disorder is a Chronic, Treatable Disease

Addiction is a stress induced genetically mediated, primary, chronic disease of brain reward, motivation, memory and related circuitry

Dysfunction in these circuits leads to characteristic biological psychological, social and spiritual manifestations

This is reflected in an individual pathologically pursuing reward and/or relief by substance use and other behaviours

# 4 ‘C’s Of Addiction

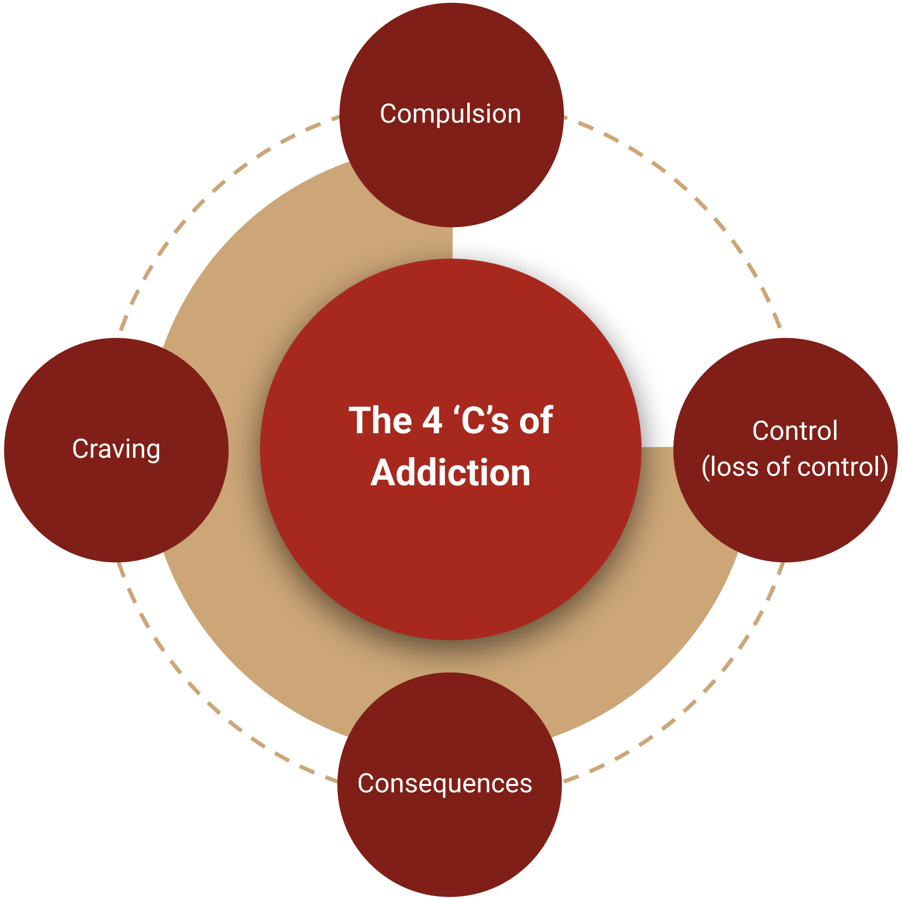

# BIOPSYCHOSOCIAL MODEL

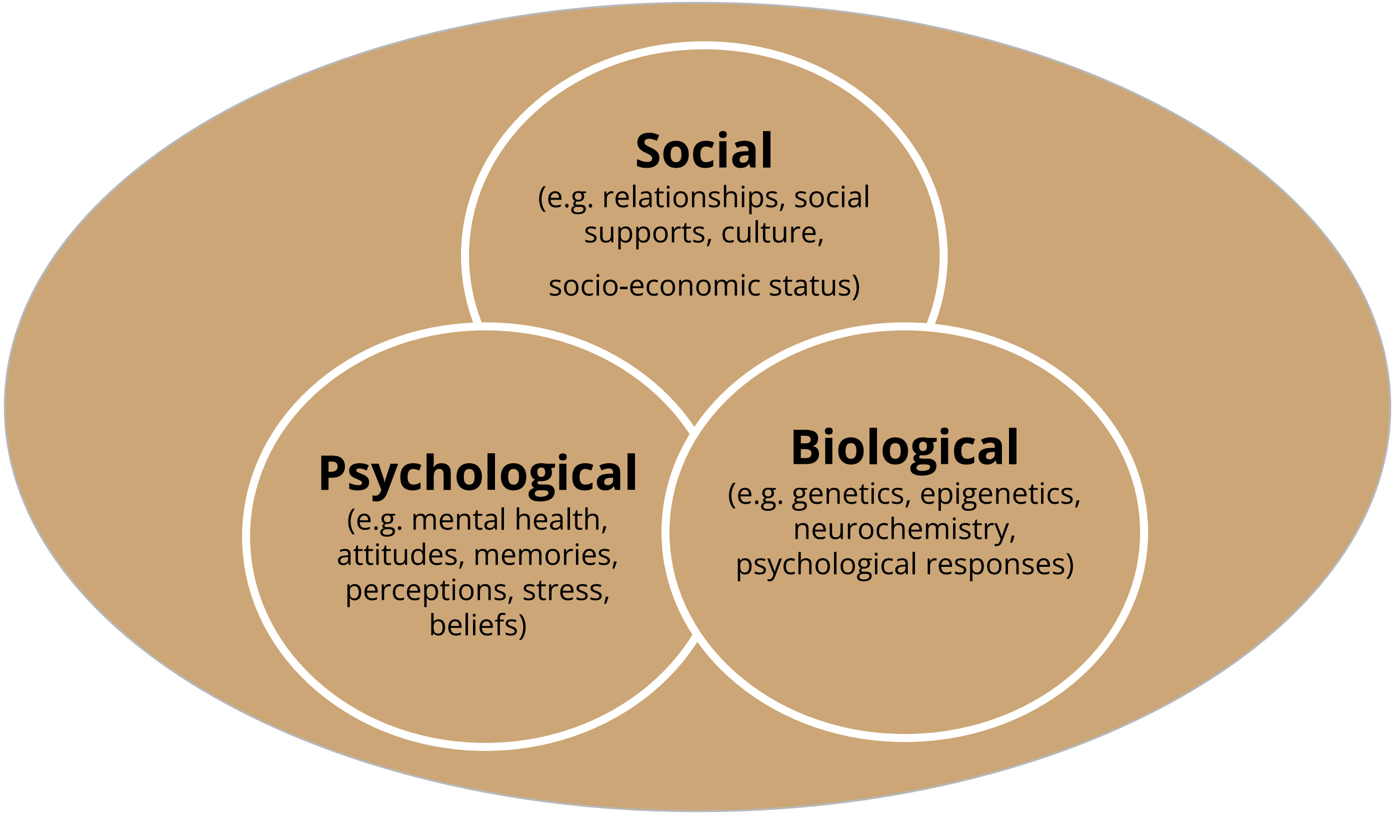

# 3 Stages of Substance Use Disorder

**Binge/Intoxication**:
the stage at which an individual consumes an intoxicating substance and experiences its rewarding or pleasurable effects

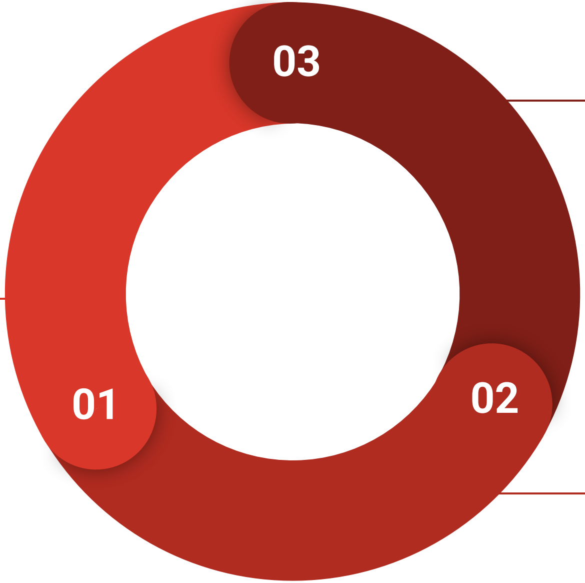


**Preoccupation/Anticipation**
the stage at which one seeks substances again after a period of abstinence..

**Withdrawal/Negative Affect**
the stage at which an individual experiences a negative emotional state in the absence of the substance; and

**The three stages are linked to and feed on each other, but they also involve different brain regions, circuits (or networks), and neurotransmitters and result in specific kinds of changes in the brain.**

# Natural Progression of Disease

**Experimentation**: Voluntary, occasional drug use without negative consequences

**Regular Use**: Increased use with higher risks of abuse, risky behaviors, and mental health issues.

**Risky Use/Abuse**: Continued use despite severe consequences, marked by cravings and withdrawal.

**Use Disorder**: Compulsive use with withdrawal symptoms and severe personal and social impacts.

# SUBSTANCE USE SPECTRUM

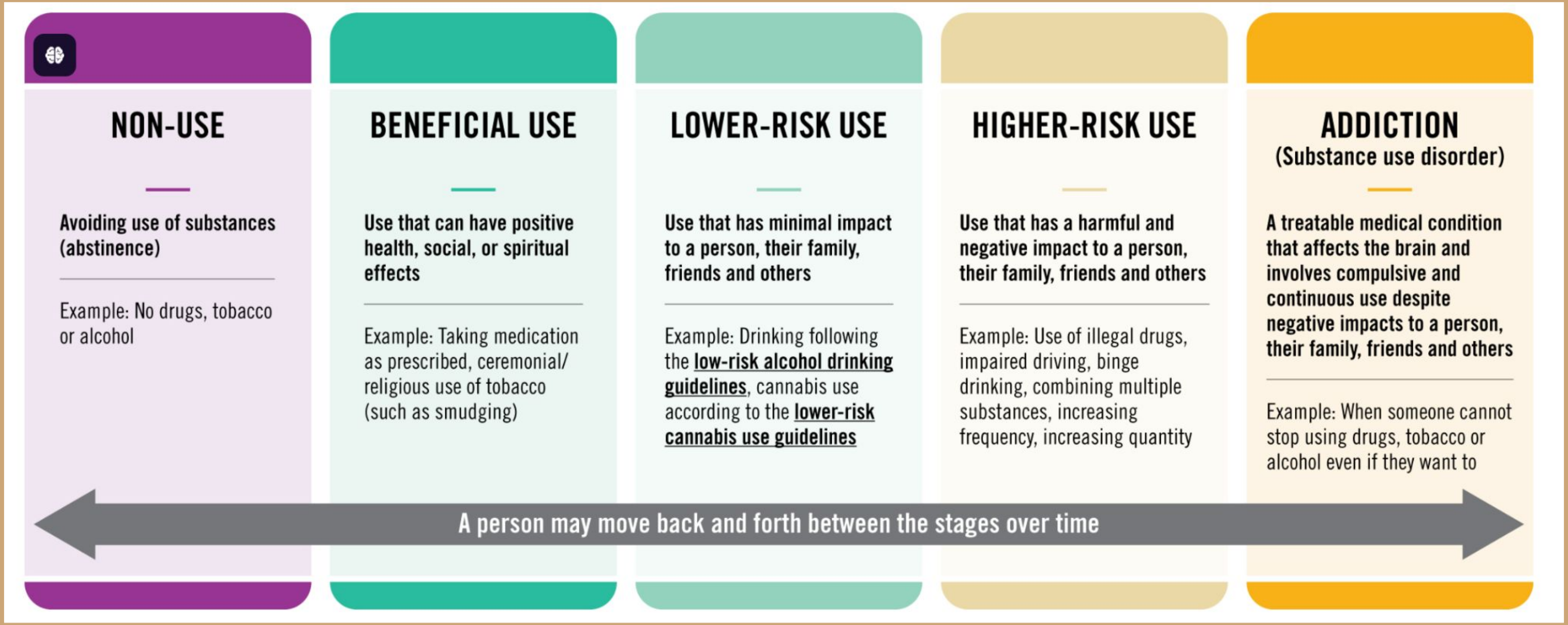


SOURCE: https://www.canada.ca/en/health-canada/services/publications/healthy-living/substance-use-spectrum-infographic.html

# WHERE DOES SBIRT FITS IN THE PREVENTION HIERARCHY

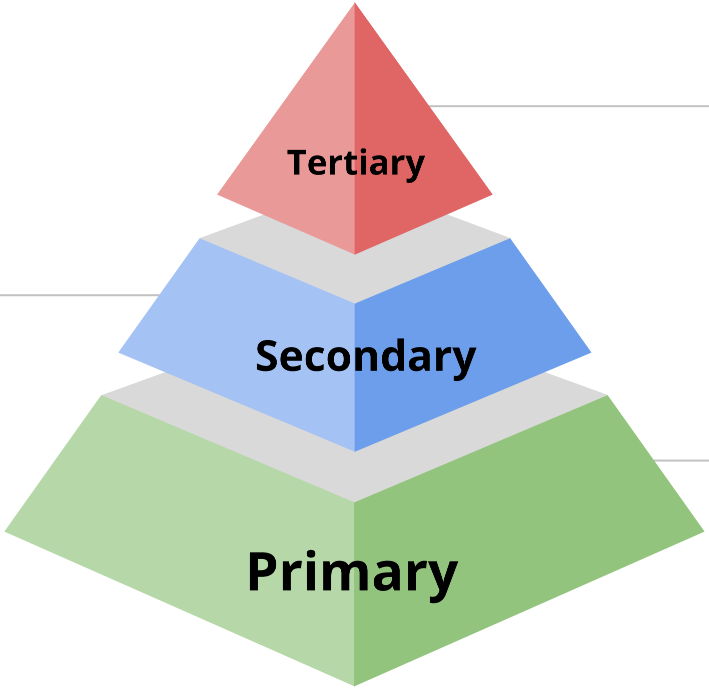


1

Crisis intervention, rehabilitation, reducing risks of health problem. **Aimed to prevent worsening conditions and the emergence of secondary problems**; *softens the impact.*

2

Aimed at changing behaviours recently initiated before they progress to sustained behaviour change, **reduces the impact.**

3

Universal interventions that address/eliminate the underlying causes of initiating harmful behaviours **before they have been contemplated or have occurred**, *focuses on prevention*

Be mindful of **health disparities** - can dramatically affect levels of screening/interventions and progression of diseases

Source: American College of Academic Addiction Medicine acaam.org

# What are the SBIRT Core Competencies?

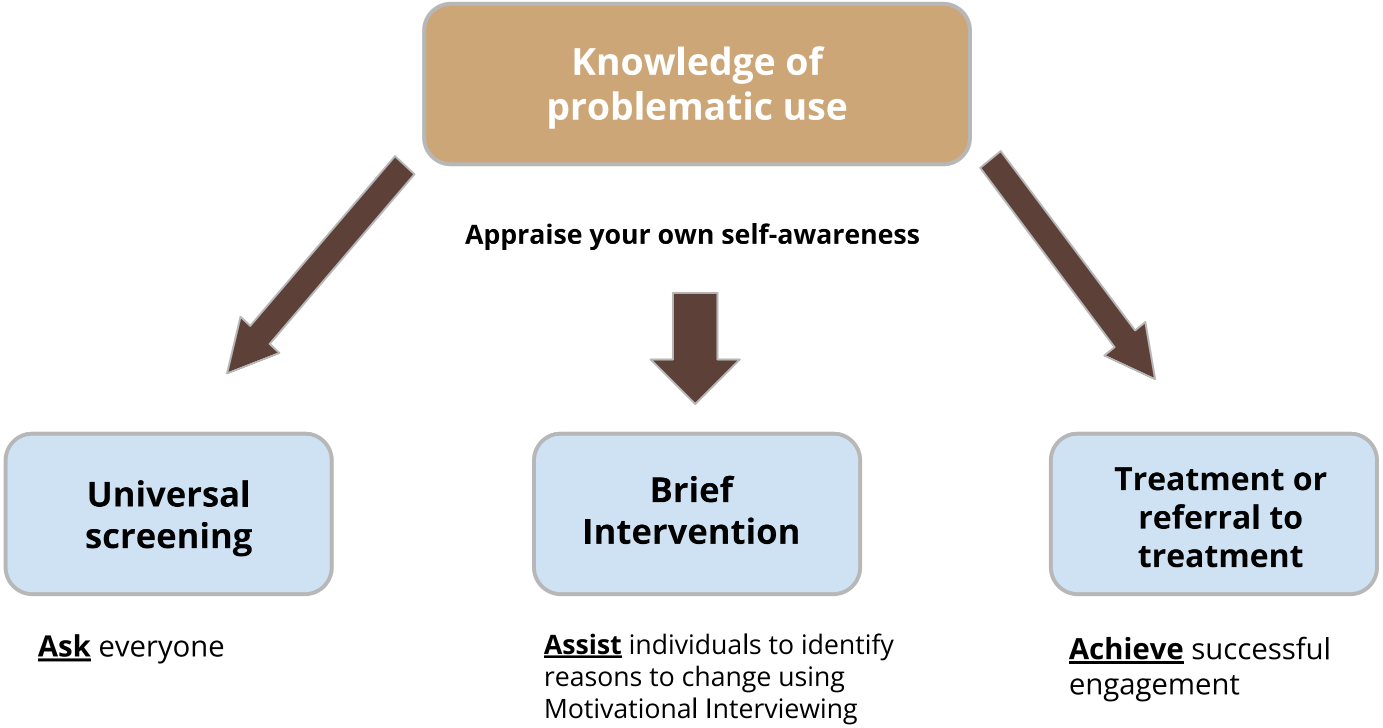

# What Does SBIRT Workflow Look Like?

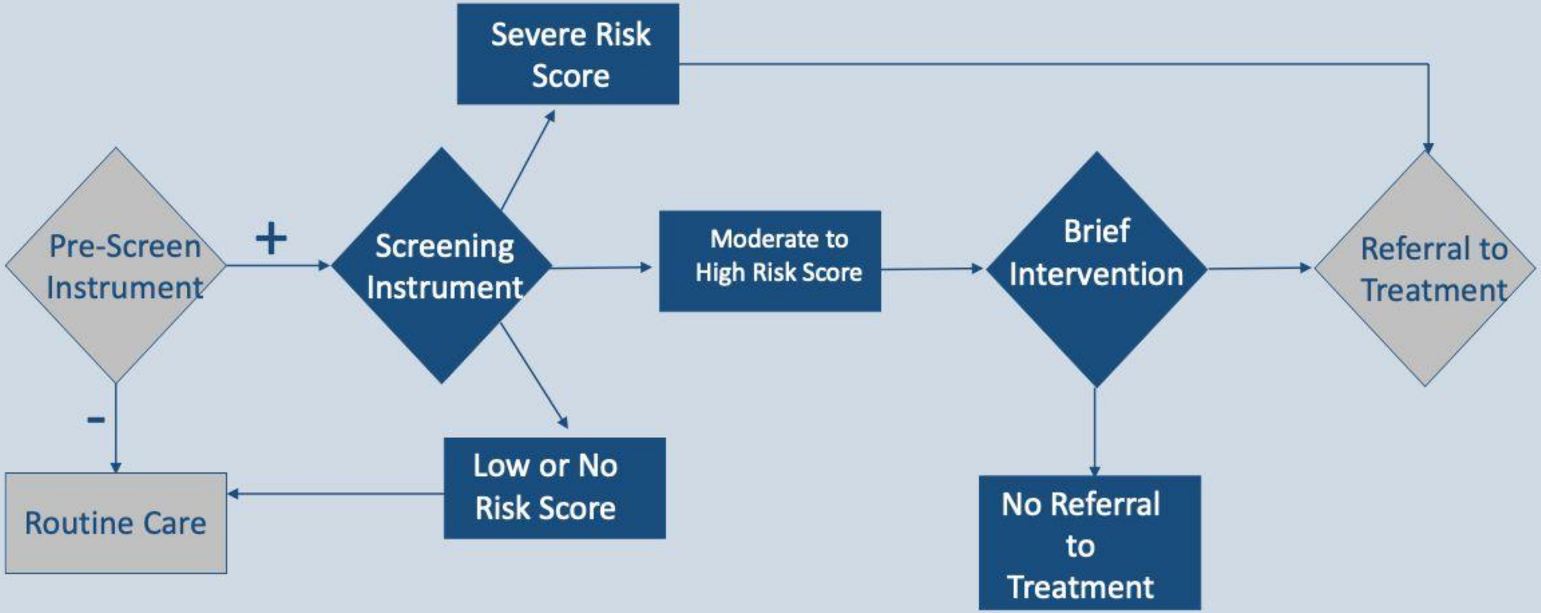


Adapted from Implementing SBIRT (Screening, Brief Intervention and Referral to Treatment) in primary care: lessons learned from a multipractice evaluation portfolio

Hargraves D, White C, Frederick R, et al. Implementing SBIRT (Screening, Brief Intervention and Referral to Treatment) in primary care: lessons learned from a multi-practice evaluation portfolio. *Public Health Reviews*. 2017;38(1). doi:10.1186/s40985-017-0077-0
Source: American College of Academic Addiction Medicine acaam.org

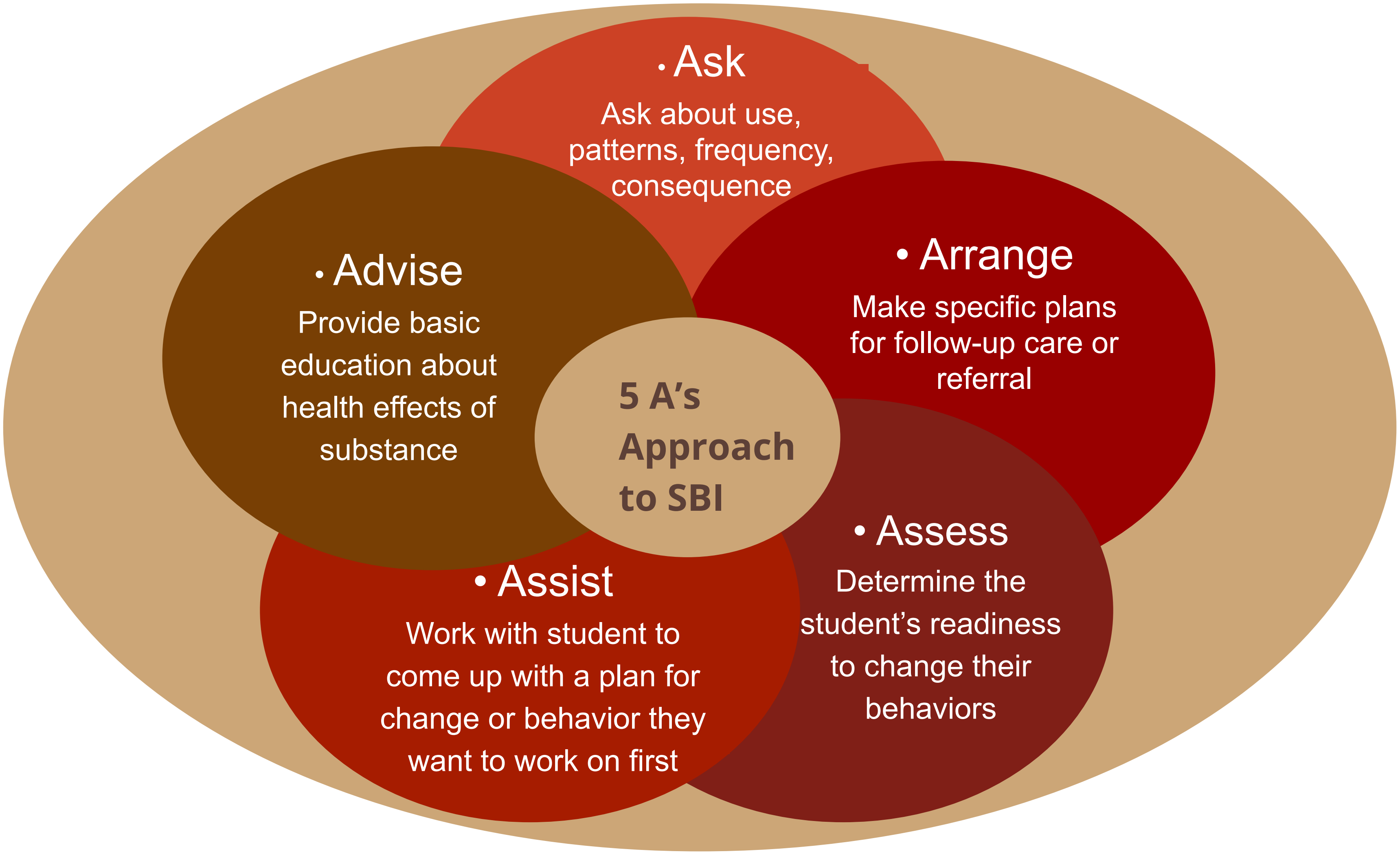
• Ask
Ask about use, patterns, frequency, consequence
• Advise
Provide basic education about health effects of substance
5 A's Approach to SBI
• Arrange
Make specific plans for follow-up care or referral
• Assess
Determine the student's readiness to change their behaviors
• Assist
Work with student to come up with a plan for change or behavior they want to work on first

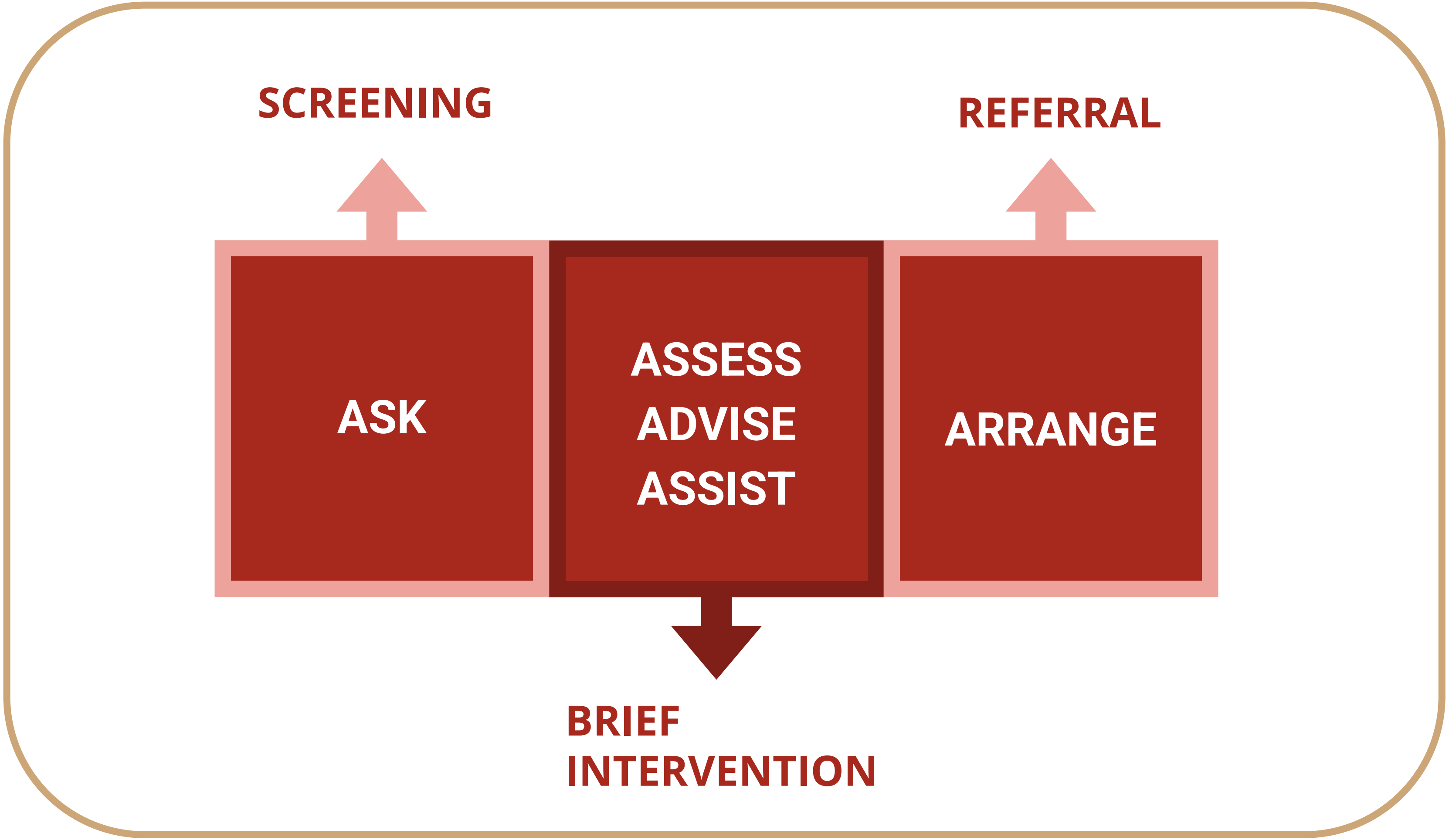
SCREENING
REFERRAL
ASK
ASSESS
ADVISE
ASSIST
ARRANGE
BRIEF
INTERVENTION

# SCREENING
# (ASK)

## GOALS OF SCREENING

- Normalizes the conversation about drug and alcohol use
- Reinforces healthy behaviors and provides opportunities for prevention
- Identifies patients who are at risk for addiction and in need of treatment for SUD
- Informs prescribing practices to avoid medication interactions and overdose
- Guides provider interventions and addiction treatment recommendations

# UNIVERSAL SCREENING WIDENS THE NET

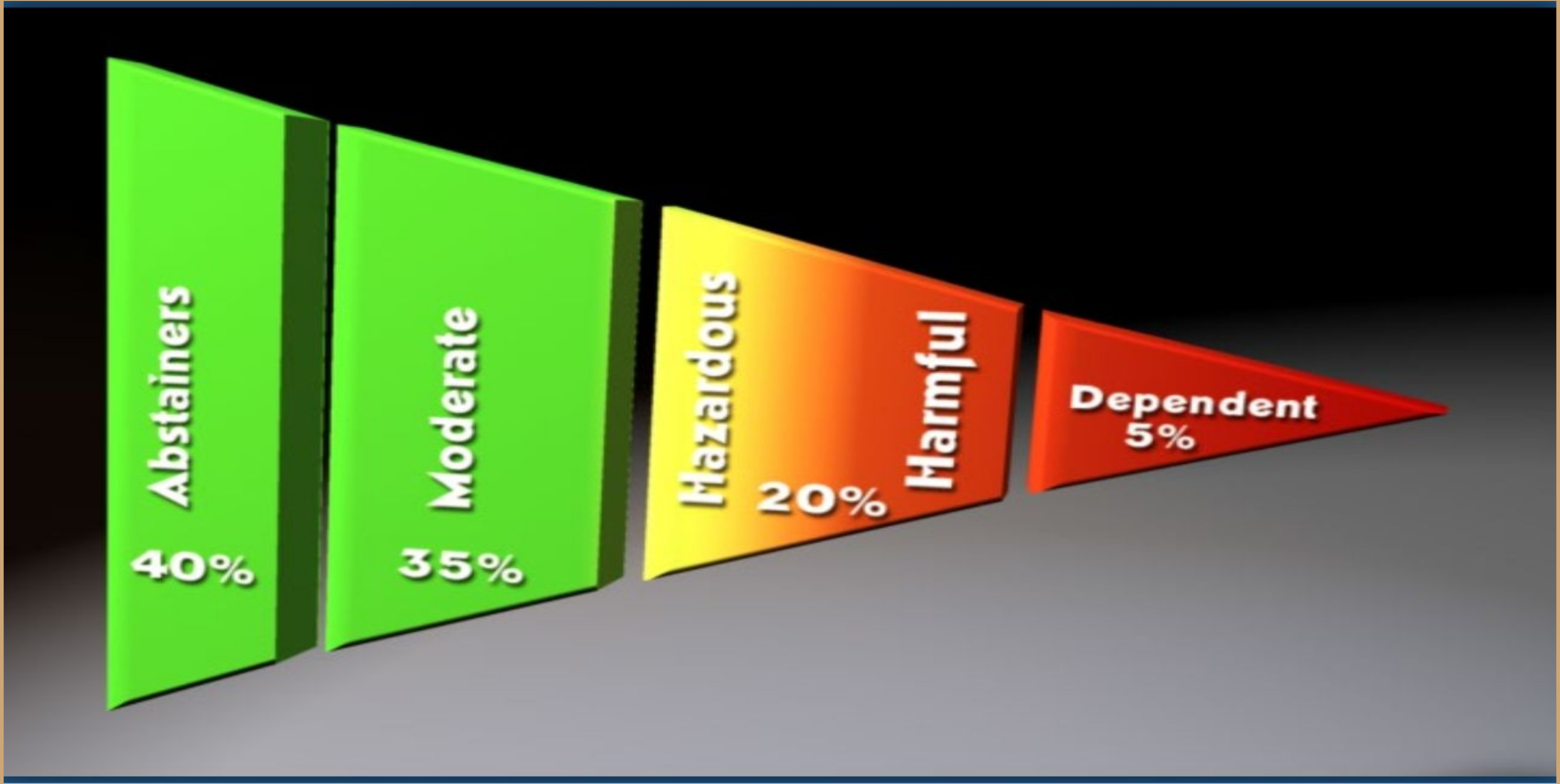


Source: American College of Academic Addiction Medicine acaam.org

# Screening Approaches and Tools:

## NIDA Approach for Adults

Unhealthy substance use screening (alcohol, drugs, tobacco)

• **Step 1** - NIDA "quick screen" - Single question about past year use:
– Tobacco Products (any use+) – proceed with quit recommendations or treatment if positive
– Alcohol (looking for heavy drinking – defined as "binge drinking")
– Illegal drugs (any use) – proceed with further screening

• **Step 2**- for those answering "yes" – do more in-depth screening or assessment to determine level of risk

# Screening Strategy for Adults: Tobacco

STEP 1: ASK - Start with single question about past year

use: Pre-screening –"quick screen"

- Tobacco – "in past year have you used any tobacco/nicotine products?"

—If YES – proceed with BI focused on cessation strategies

—Quit recommendations or treatment

# Screening: ALCOHOL

**STEP 1 - ASK** – "do you sometimes drink beer, wine, or other alcoholic beverages?"

• If yes, ask:

– In the past year how many times have you had 5+ (4+ for females) drinks in a day?

– If YES – screen is considered positive: 81.8% sensitivity; 79.3% specificity for detection of AUD.

– Needs further assessment about consequences.

– If NO – Step 2

**STEP 2: FURTHER ASSESS of USE:** determine level of risk:

– Ask frequency and quantity questions from NIAAA.

◦ On average, how many days per week do you drink alcohol?

◦ On a typical day when you drink, how many drinks do you have?

◦ What's the maximum number of drinks you have had on a given occasion in the last month?

◦ Determines whether at-risk or heavy drinker needing further in-depth assessment or brief intervention

# A STANDARD DRINK

**Nigeria is in dire need to develop a standard drink measure and low drinking guidelines**

**According to the WHO, standard drink measures the amount of pure alcohol consumed, usually between 8 g and 12 g**

What is a standard drink? This is equivalent to a unit of alcohol

- ½ bottle of standard beer
- 300 - 400 ml standard beer
- ¼ bottle strong beer
- ½ packet of Locally brewed gin
- 30 ml of spirits (whisky, gin, vodka)

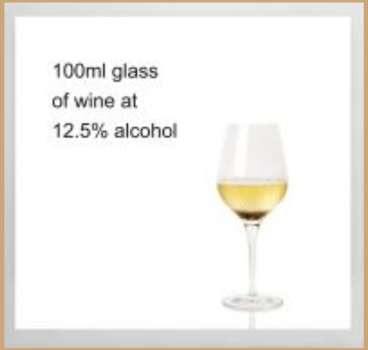


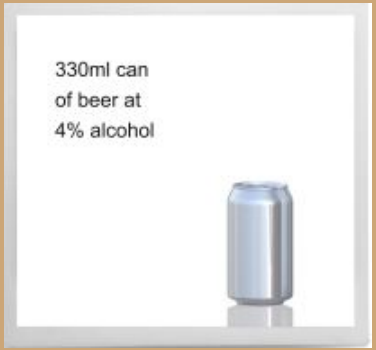

# NIAAA Standard for AT-risk Drinking

| | Drinks per week | Drinks per occasion |
|---|---|---|
| **Men** | >14 | >4 |
| **Women** | >7 | >3 |
| **All Age >65** | >7 | >3 |

# COMMON BEER BRANDS IN NIGERIA AND THEIR ALCOHOL COMPOSITION

| Beer brand | Bottle size | Alcoholic concentration | Total alcohol content (grams) | Units |
|---|---|---|---|---|
| Heineken lager | 600ml (bottle)<br>330ml (can) | 5% | 23.7g<br>13g | 3.0<br>1.7 |
| Star Radler | 450ml (bottle)<br>330cl (can) | 2% | 7.1g<br>5.2g | 0.9<br>0.7 |
| Smirnoff Ice | 600ml (big bottle)<br>330ml (Small bottle) | 4% | 18.9g<br>10.4g | 2.4<br>1.3 |
| Star lager | 600ml | 5.1% | 24.2g | 3.1 |
| Origin | 200ml (Bitters)<br>600ml (Beer) | 30%<br>6% | 47.3g<br>28.4g | 6.0<br>3.6 |
| Guinness stout | 600ml (Big bottle)<br>325ml (Small bottle) | 7.5% | 35.6g<br>19.3g | 4.5 |
| Trophy | 600ml | 5.2% | 24.6g | 3.1 |

# OTHER COMMON BEER BRANDS IN NIGERIA AND THEIR ALCOHOL COMPOSITION

| Beer brand | Bottle size | Alcoholic concentration | Total alcohol content (grams) | Units |
|---|---|---|---|---|
| Legend stout | 600ml (big bottle)<br>325ml (Small bottle) | 7.5% | 35.6g<br>19.3g | 4.5<br>2.4 |
| Harp lager | 600ml | 5.15% | 24.4g | 3.1 |
| Gulder lager | 600ml | 5.2% | 24.6g | 3.1 |
| 33 lager | 600ml | 5.2% | 24.6g | 3.1 |
| Life lager | 600ml | 5.0% | 23.7g | 3.0 |

# According to WHO, no level of alcohol consumption is safe for our health

Anderson BO, Berdzuli N, Ilbawi A, et al. Health and cancer risks associated with low levels of alcohol consumption. *The Lancet Public Health*. 2023;8(1):e6-e7. doi:10.1016/s2468-2667(22)00317-6

# FOR FURTHER SCREENING: ALCOHOL

For further screening and assessment can proceed with:

— AUDIT, AUDIT-C, CAGE, TAPS screener, NIDA- Modified ASSIST

— Pregnant women:

- T-ACE: Tolerance, Annoyed, Cut Down, Eye-opener
- TWEAK: Tolerance, Worry, Eye-opener, Amnesia, Kut Down
- 5Ps - Parents, Peers, Partner, Pregnancy, Past

— Children:

- Single question screeners for drugs and Alcohol - S2BI, BSTAD, NIAAA
- CRAFFT, DAST 20, AUDIT C

- **Information from these leads to Step 3 – ADVISE, ASESS  AND ASSIST (Brief intervention)**

# AUDIT – Alcohol Use Disorders Identification Test

• Developed by WHO to assess drinking behaviors and problems

**• 10 questions:**

— How often do you have a drink containing alcohol

— How many drinks containing alcohol do you have on a typical day when you are drinking?

— How often do you have 6 or more drinks on one occasion?

— How often during the last year have you found that you were not able to stop drinking once you had started?

— How often during the last year have you failed to do what was normally expected of you because of drinking?

— How often during the last year have you needed a first drink in the morning to get your yourself going after a heavy drinking session?

— How often during the last year have you had a feeling of guilt or remorse after drinking?

— How often during the last year have you been unable to remember what happened the night before because of your drinking?

— Have you or someone else been injured because of your drinking?

— Has a relative, friend, or doctor, or other health care worked been concerned about your drinking or suggested you cut down?

**• Scored 0-4: never, monthly or less, 2-4 times a month, 2-3 times a week, 4+ times a week**

Bohn MJ, Babor TF, Kranzler HR. The Alcohol Use Disorders Identification Test (AUDIT): validation of a screening instrument for use in medical settings. *Journal of Studies on Alcohol*. 1995;56(4):423-432. doi:10.15288/jsa.1995.56.423

# AUDIT Scoring

Positive Score =

- >8 = harmful or hazardous use
- >13 for female or >15 for males – consistent with AUD

Bohn MJ, Babor TF, Kranzler HR. The Alcohol Use Disorders Identification Test (AUDIT): validation of a screening instrument for use in medical settings. *Journal of Studies on Alcohol*. 1995;56(4):423-432. doi:10.15288/jsa.1995.56.423

# Pre Screening for Drugs : NIDA single Question

**"How many times in the past year have you used an illegal drug or a prescription medication for non-medical reasons?"**

**(...for instance, because of the feeling it caused or experience you have..)**

- If response is, "None," screening is complete.
- If response is positive, inquire further - using screening tools

**Sensitivity/Specificity: 100% / 74% for a drug disorder**

Source: Smith, P.C., Schmidt, S.M., Allensworth-Davies, D., & Sgitz, R. (2010). A single-question screening test for drug use in primary care. Arch Intern Med, 170(13), 1155-1160

# FURTHER SCREENING TOOLS: DRUGS

- NM- ASSIST – Alcohol, Smoking and Substance Involvement Screening test
- DAST – Drug Abuse Screening Test (excludes alcohol, Tobacco)
- CAGE-AID – Adapted to Include Drugs*
- **TAPS – Tobacco, Alcohol, Prescription Drug misuse, and other Substances screener**

- Note: no screening tests have been validated for universal screening for prescription drug misuse in primary care settings.

# NIDA-Modified (NM) Assist Screening Tool

- In the past year, how often have you used the following?

– Alcohol (focuses only on binge drinking levels)
– Tobacco products
– Prescription drugs for non-medical reasons
– Illegal drugs
◦ Cannabis
◦ Cocaine
◦ Stimulants
◦ Meth
◦ Inhalants
◦ Sedatives
◦ Hallucinogen
◦ Street opioids
◦ Prescription opioids

— Answers: Never, Once or Twice, Monthly, Weekly, Daily or Almost daily

- For each positive: Strong desire to use, leads to problems, Fail to meet expectations, Relatives expressing concerns about use, Failure to cut down or quit, Injection use.

Oga EA, Mark K, Peters EN, Coleman-Cowger VH. Validation of the NIDA-modified ASSIST as a screening tool for prenatal drug use in an urban setting in the United States. *Journal of Addiction Medicine*. 2020;14(5):423-430. doi:10.1097/adm.0000000000000614

# NIDA-Modified (NM) Assist Screening Tool

Provides score for each substance, and recommendations, educational info

• Can be used for alcohol, tobacco, illegal substances, non-medical use of Rx drugs

—Low 0-3

• Provide feedback, reinforce abstinence, offer continuing support

—Moderate 4-26

• Provide feedback on screening results, ADVISE, ASSESS and ASSIST, consider referral based on clinical judgement, offer continuing support

—High >26

• Provide feedback on screening results, ADVISE, ASSESS, and ASSIST, arrange referral, offer continuing support

Oga EA, Mark K, Peters EN, Coleman-Cowger VH. Validation of the NIDA-modified ASSIST as a screening tool for prenatal drug use in an urban setting in the United States. *Journal of Addiction Medicine*. 2020;14(5):423-430. doi:10.1097/adm.0000000000000614

# TAPS – From NIDA Clinical Trials Network

Part 1:

– 4 Questions related to Tobacco, Alcohol, any Illicit drug use (includes marijuana) and Prescription medication misuse

– Used assessed over previous 12 months

– Scored from monthly to daily/almost daily

• Part 2

– 9 questions about use over previous 3 months

– Tobacco, alcohol, concern over drinking, marijuana, cocaine, heroin, opiate pain meds, medications for anxiety or sleep, misuse of medication for ADHD, “any other recreational drug”

– Yes/No answers

McNeely J, Wu L, Subramaniam G, Sharma G, Cathers LA, Svikis D, et al. Performance of the Tobacco, Alcohol, Prescription Medication, and Other Substance Use (TAPS) Tool for Substance Use Screening in Primary Care Patients. Ann Intern Med. 2016;165:690-699. doi: 10.7326/M16-031

# Scoring of TAPS

- Part 1:

– Positive = One any answer other than “never” on each of 4 questions

- Part 2:

– Designed to assess risk level – from “problem use” to SUD

◦ 0 – no use in previous three months

◦ 1 – Problem use

◦ 2+ - Higher risk - consistent with SUD

– Sensitivity >70% for tobacco, alcohol and marijuana for score 2+;

◦ Lower for other substances, suggested to have lower level of suspicion for higher risk assessment.

McNeely J, Wu L, Subramaniam G, Sharma G, Cathers LA, Svikis D, et al. Performance of the Tobacco, Alcohol, Prescription Medication, and Other Substance Use (TAPS) Tool for Substance Use Screening in Primary Care Patients. Ann Intern Med. 2016;165:690-699. doi: 10.7326/M16-031

# SCREENING TOOLS FOR ADOLESCENTS

# PRE-SCREEN

S2BI—Screening to Brief Intervention : asks a **single frequency question** for past year use of the three substances most commonly used by adolescents: tobacco, alcohol, and marijuana.

If positive, screens further  for prescription drugs, illegal drugs, inhalants and synthetic drugs.

• Discriminates among 4 categories of substance use experience:
–No past-year use – No use
–Use without a SUD – Once or twice use of any substance
–Mild-moderate SUD – Monthly use
–Severe SUD – Weekly or greater use

• Sensitivity – 100%; Specificity – 84% for non-tobacco substance use –90% and 94% for SUD; 100% and 94% for severe SUD; 75% and 95% for nicotine dependence

• Other pre-screen tools;
—BSTAD; Brief Screener for Tobacco, Alcohol and Other Drugs
—NIAAA; 2 question screener for Alcohol

Levy S, Weitzman ER, Marin AC, Magane KM, Wisk LE, Shrier LA. Sensitivity and Specificity of S2BI for Identifying Alcohol and Cannabis use Disorders among Adolescents Presenting for Primary Care. *Substance Abuse*. 2020;42(3):388-395. doi:10.1080/08897077.2020.1803180

# FURTHER SCREENING TOOLS FOR ADOLESCENTS

For Further Assessment,

- CRAFFT Tool is validated for adolescents and young adults within ages 12-21.
- Screens alcohol, cannabis, and other drugs including vaping devices containing nicotine and other products.
- Can be self and clinician administered
- Has 3 parts A, B, C

Mitchell SG, Kelly SM, Gryczynski J, et al. The CRAFFT Cut-Points and DSM-5 Criteria for Alcohol and Other Drugs: A Reevaluation and Reexamination. *Substance Abuse*. 2014;35(4):376-380. doi:10.1080/08897077.2014.936992

# CRAFFT TOOL

**PART A:** ask past year use of alcohol, marijuana, and other drugs.
If **Part A = 0**, ask **only the first question** in Part B. If **Part A ≥ 1**, ask **all six questions** in Part B

**PART B:**
**C**—Have you ever ridden in a **CAR** driven by someone (including yourself) who was "high" or had been using alcohol or drugs?
**R**—Do you ever use alcohol or drugs to **RELAX**, feel better about yourself, or fit in?
**A**—Do you ever use alcohol or drugs while you are by yourself, or **ALONE**?
**F**—Do you ever **FORGET** things you did while using alcohol or drugs?
**F**—Do your **FAMILY or FRIENDS** ever tell you that you should cut down on your drinking or drug use?
**T**—Have you ever gotten into **TROUBLE** while you were using alcohol or drugs?

**PART C: ask vaping devices containing nicotine and/or flavors, or use of any tobacco products.** If **Part C ≥ 1suggests problem, requiring further assessment**

Mitchell SG, Kelly SM, Gryczynski J, et al. The CRAFFT Cut-Points and DSM-5 Criteria for Alcohol and Other Drugs: A Reevaluation and Reexamination. *Substance Abuse*. 2014;35(4):376-380. doi:10.1080/08897077.2014.936992

# CRAFFT Scoring

Each “yes” = 1 point

**Negative CRAFFT Screen (score of 0 or 1)**

- Score 0=Praise and encourage safe behavior
- Score 1 = “You have made some smart decisions, (give example), but I am concerned about ….”
  - Encourage abstinence.

**Positive CRAFFT Screen (score of 2 or greater)**

- Adolescent is at high risk
- Further questioning
- Rule out SUD

Mitchell SG, Kelly SM, Gryczynski J, et al. The CRAFFT Cut-Points and DSM-5 Criteria for Alcohol and Other Drugs: A Reevaluation and Reexamination. *Substance Abuse*. 2014;35(4):376-380. doi:10.1080/08897077.2014.936992

# USING THE 5 R'S BRIEF COUNSELING

1. **Review**: Discuss screening results. For each "yes," ask, *"Can you tell me more about that?"*
2. **Recommend**: Advise against alcohol, nicotine, or drug use, highlighting risks like brain harm, memory issues, and dangerous situations.
3. **Riding/Driving**: Counsel on risks of motor vehicle crashes and encourage a family safety plan.
4. **Response**: Elicit motivation with questions like, *"What are the benefits of not using?"*
5. **Reinforce**: Encourage self-efficacy by affirming their ability to achieve goals without substance use.

**Provide a Contract for Life** (available at www.crafft.org/contract)

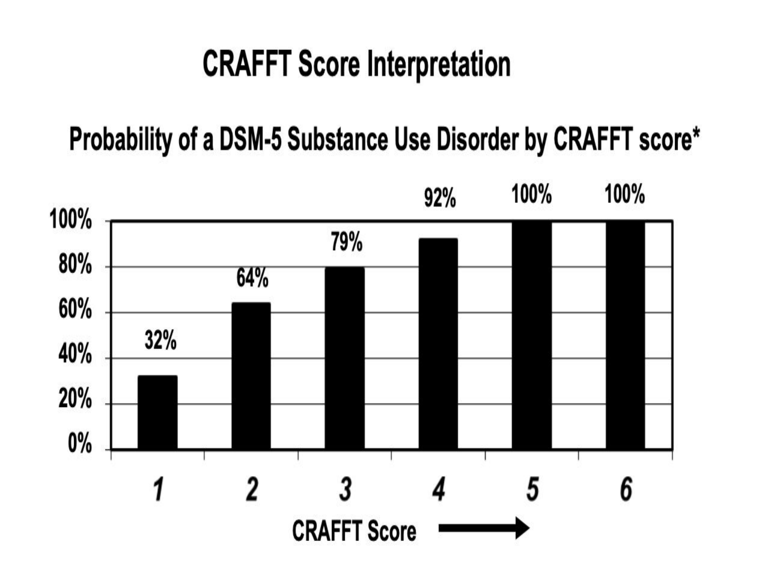


Mitchell SG, Kelly SM, Gryczynski J, et al. The CRAFFT Cut-Points and DSM-5 Criteria for Alcohol and Other Drugs: A Reevaluation and Reexamination. *Substance Abuse*. 2014;35(4):376-380. doi:10.1080/08897077.2014.936992

# BRIEF INTERVENTIONS (ADVISE, ASSESS, ASSIST)

# Brief Interventions(BI)

Clinical strategy designed to change behavior

- Not using – support abstinence
- Risky Use – goal is to reduce use/abstain
- Addiction – goal is to agree to referral or treatment

Evidence-based from research studies – motivational interviewing

- Effective for tobacco/nicotine, alcohol & other drugs
- Only 5-15 minutes needed per patient encounter

# What does research tell us about BI

## Brief Interventions (BI) in a Primary Care setting

- Are low cost and effective, particularly for alcohol misuse
- By intervening early, SBIRT saves lives and money, and is consistent with overall support for patient wellness

# What to do with a positive response from a patient?

**Use Motivational Interviewing (MI), a collaborative, patient-centered approach to strengthen motivation for change(Millner & Rollnick, 2009)**

**MI focuses  on evoking motivation, encouraging change talk, negotiating a treatment plan if the patient is ready, and leveraging their strengths/strategies.**

- ***How ready is the patient to change behavior on a scale of 1-10 (readiness ruler)? Is he/she interested in setting a goal for reduction or elimination of use? What things has he/she considered trying?***

# Motivational Interviewing is a.....

**person-centered**, **collaborative conversation** style that…

- ➔ explores and resolves **ambivalence** about change
- ➔ enhances a person’s **intrinsic motivation**
- ➔ strengthens **commitment** to change
- ➔ involves a **change goal** (target behavior)
- ➔ pays careful attention to **language** of change

-William Miller and Steven Rollnick, 2013

# MI Core Communication Skills

**OARS**

- **O**pen questions
- **A**ffirmations
- **R**eflections
- **S**ummaries

+

**EPE**

Providing information and advice with permission (Elicit-Provide-Elicit)

# General Principles for Behaviour Change

- **Respect for autonomy** of patients and their choices
- Readiness to change must be taken into account
- **Ambivalence** is common
- **Goals selected by the patient**, not the expert
- Expert is the provider of the information
- Patient is the active decision-maker

# ADVISE, ASSESS, ASSIST – BI

**ADVISE**

- **State Recommendations Clearly**: Share conclusions and suggestions in a supportive, straightforward manner

**ASK**

- **Gauge Readiness**: Ask, *“Are you open to making changes to your substance use habits?”*

**ASSESS**

- **Plan Next Steps for AUD/SUD**: Refer for evaluation, suggest mutual help groups, or recommend withdrawal management/medications as needed.
- **Support Commitment**: Help motivated patients set goals, create a plan, and provide educational resources.
- **Stay Encouraging**: For unready patients, express concern, encourage reflection, and reaffirm your willingness to help when they’re ready.

# REFERRAL TO TREATMENT (ARRANGE)

# ARRANGE: providing continuing support

**Provide follow-up appointment or prompt referral when appropriate**

When seen in follow-up, determine if patient is able to meet or sustain their selected goals

- If yes, reinforce and support commitment, encourage follow-up.
- If No, acknowledge change is difficult, normalize the conversation, ambivalence is common, negotiate goals and plan, consider engaging others, reassess.

# REFERRAL

- Refer patients to **addiction counselors,, addiction physicians,, mental health professionals (psychologists/psychiatrists), social workers**, **peer recovery coaches, harm reduction specialists**

- Discharge sheet of possible centers and / or programs and information

# BRIEF NEGOTIATED INTERVIEW (BNI)

Ok, So now we know the 5A's approach of SBIRT

What is the Brief Negotiated Interview & How do I perform this technique?

## BNI: Brief Negotiation Intervention

Developed by team at Yale Department of Emergency Medicine

- Short (5-7 min) counseling sessions

Based on motivational interviewing techniques

- patient-centered
- use of reflective listening
- assists in changing specific drinking/drug-using habits

# COMPONENTS OF BNI

1. Raise the subject
2. Provide Feedback
3. Enhance Motivation
4. Negotiate And Advise

# STEP 1: RAISE THE SUBJECT

**First, establish rapport with the patient**

- ➔ Appreciate their perspectives
- ➔ Explain your concerns & your role
- ➔ Avoid judgment & blame

# STEP 1: RAISE THE SUBJECT

**Discussing Substance Use**

- ➔ Seek permission to start the conversation.
- ➔ Agree to explore the topic together.
- ➔ Discuss the pros and cons of substance use.
- ➔ Summarize and reflect on the pros and cons shared by the patient.

# STEP 2: PROVIDE FEEDBACK

**Express empathy**

- Review results of screen
- Make connection to any medical consequence
- Provide education about effects of substances used

# STEP 2: PROVIDE FEEDBACK

Provide information about risks of specific substance being used.

Tailor to specific substance, level of use and consequences already experienced

> "This is what we know about the risks of X (i.e., alcohol, marijuana, etc.) for your age."

> "These are the possible health risks you may experience because of your use."

# Step 3: Enhance Motivation

**Assess readiness to change**

"On a scale of 1-10 (1 being not ready and 10 being very ready) how ready are you to change any aspect your drinking /drug use?"

| | | | | | | | | | |
|---|---|---|---|---|---|---|---|---|---|
| 1 | 2 | 3 | 4 | 5 | 6 | 7 | 8 | 9 | 10 |

# Step 3: Enhance Motivation

**Develop discrepancy**

- Identify areas to discuss
- Explore pros and cons if readiness to change is low
- Use reflective listening

If patient indicates:

- > 2 : “Why did you choose that number and not a lower one?”

“What are some reasons why you are thinking about changing?”

- < 1: “What would make this a problem for you?”

“Have you ever done anything that you wish you hadn’t while drinking/using the substance?”

# Step 4: Negotiating a Treatment Plan

Build upon a patient's readiness and strengths and follow up on statements about willingness to take action.

**Next steps need to come from your patient.**

*"I will go to a SMART Recovery Meeting tomorrow" (Commitment)*

*"I've started taking a medication to help me avoid relapse to heroin." (Taking steps)*

*"I am prepared to go to counseling once week" (Activation)*

# Common Mistakes to avoid Negotiating a Treatment Plan

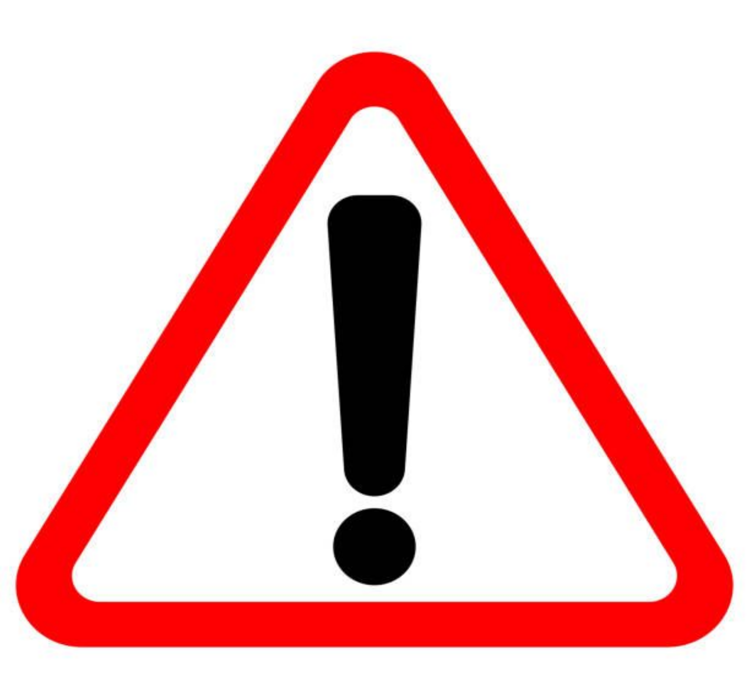

- Avoid rushing to create a treatment plan if the patient isn't ready.
- Consider cultural factors that may affect treatment acceptance.
- Incorporate pharmacotherapy to support recovery.
- Ensure referrals are to appropriate, accessible programs.
- View the patient as managing a chronic disease, not as resistant or self-sabotaging.

# STIGMA AND DISCRIMINATION IN CARE SETTINGS

**What is stigma?**: Stigma involves discrimination and unfounded beliefs about people with SUD, such as being dangerous, at fault, or unable to manage treatment.

**Where does stigma come from?**: Stigma often stems from outdated views of addiction as a moral failing rather than a chronic, treatable disease.

**How does stigma affect people with SUD?**: Stigma discourages individuals from seeking treatment, fosters negative stereotypes, and impacts the quality of care provided by healthcare professionals.

**How can we change stigmatizing behavior?**: Use non-stigmatizing, person-first language that reflects scientific understanding and avoids equating individuals with their condition.

**What else should I keep in mind?**: Use “substance use” for all substances, specify severity levels (e.g., mild, moderate, severe), and employ evidence-based language when discussing treatment plans.

# Destigmatizing Language in care settings - Word Matters

| INSTEAD OF… | USE …… | BECAUSE…… |
| --- | --- | --- |
| **Addict** | Person with substance use disorder | Person-first language<br><br>The change shows that a person has a problem, rather than is the problem<br><br>The terms avoid eliciting negative connotations and individual blame. |
| **User** | Person with OUD or person with opioid addiction (when substance in use is opioids) | |
| **Junkie** | Person in active use; use the person's name, and then say "is in active use. | |
| **Alcoholic** | Person with alcohol use disorder | |
| **Drunk** | Person who misuses alcohol/engages in unhealthy/hazardous alcohol use | |
| **Former Addict** | Person in Recovery | |

Words matter: preferred language for talking about addiction | National Institute on Drug Abuse. National Institute on Drug Abuse. Published November 18, 2024.
https://nida.nih.gov/research-topics/addiction-science/words-matter-preferred-language-talking-about-addiction

# Destigmatizing Language in care settings - Word Matters

| INSTEAD OF… | USE …… | BECAUSE…… |
|---|---|---|
| Abuse | **For illicit drugs:**<br><br>Use<br>**For prescription medications:**<br><br>Misuse<br>Used other than prescribed | The term "abuse" was found to be associated with negative judgments and punishment.<br><br>Prescription medications should only be used as directed by the person they are prescribed for; any other use is considered misuse. |
| Clean<br><br>Dirty | **For toxicology screen results:**<br>Testing negative<br><br>**For non-toxicology purposes:**<br>- Being in remission or recovery<br>- Abstinent from drugs<br>- Not drinking or taking drugs<br>- Not currently or actively using drugs | Use accurate, non-stigmatizing language as you would for any medical condition.<br><br>Set an example by avoiding stigmatizing slang when treating patients.<br><br>Stigmatizing terms can trigger negative and punitive biases. |

Words matter: preferred language for talking about addiction | National Institute on Drug Abuse. National Institute on Drug Abuse. Published November 18, 2024.https://nida.nih.gov/research-topics/addiction-science/words-matter-preferred-language-talking-about-addiction

# Destigmatizing Language in care settings - Word Matters

| INSTEAD OF… | USE …… | BECAUSE…… |
|---|---|---|
| • Opioid substitution replacement therapy<br>• Medication-assisted treatment (MAT) | • Opioid agonist therapy<br>• Pharmacotherapy<br>• Addiction medication<br>• Medication for a substance use disorder<br>• Medication for opioid use disorder (MOUD | Medications don't "substitute" one drug or addiction for another; this is a misconception.<br><br>The term MOUD (Medications for Opioid Use Disorder) reflects their central role in treatment, similar to antidepressants or antipsychotics, unlike the term MAT, which implies a temporary or supplemental role. |

Words matter: preferred language for talking about addiction | National Institute on Drug Abuse. National Institute on Drug Abuse. Published November 18, 2024.https://nida.nih.gov/research-topics/addiction-science/words-matter-preferred-language-talking-about-addiction

# SUMMARY

- Substance use disorders are **common, identifiable, and treatable health conditions.**
- **Early detection and intervention** can significantly improve outcomes.
- Use a **validated tool**
- Incorporate as routine part of another health screening to reduce **stigma**
- Explore each substance - **many patients use more than one**
- Use **motivational interviewing skills,** compassionate and **nonjudgmental approach** fosters trust and encourages patient engagement.

# REMEMBER…….

Just start the conversation,

you may save a life!

# PARTNERS

NIGERIAN MEDICAL ASSOCIATION 1960 LAGOS STATE

NATIONAL AGENCY FOR FOOD AND DRUG ADMINISTRATION AND CONTROL NAFDAC

ISSUP INTERNATIONAL SOCIETY OF SUBSTANCE USE PROFESSIONALS NIGERIA chapter

LAGOS STATE JUSTICE AND PROGRESS Lagos State HSC

Resurgence Mental Health Support Initiative

NDLEA NATIONAL DRUG LAW ENFORCEMENT AGENCY

SMART Recovery Life beyond addiction NIGERIA

# SBIRT Volunteers

- ★ Lucky Success
- ★ Anethua Justina
- ★ Chinyere Nduu Okoro
- ★ Ifechi Ugwuibe
- ★ Bekederemo Angela
- ★ Dr. Ayo Adeniji
- ★ Joy M Gurindapalli
- ★ Nehemiah Samuel
- ★ Angela Bekederemo
- ★ Olusesan Samuel-Kayode
- ★ Justice Anaba
- ★ Dr. Nkemakolam Ndionuka
- ★ Dr. Adetolu Isaac Olatunde
- ★ Titilope Monsurat Ogunluyi

We are grateful for all your contributions to the success of the SBIRT Training for Lagos state primary care physicians..

# Thank You for your support!

# SUBSTANCE USE TREATMENT CENTRES IN LAGOS STATE

| Name | Address | Contact Person | Contact Number | Contact Email | Fee Status |
|---|---|---|---|---|---|
| Federal Neuro Psychiatric Hospital, (FNPH) Yaba, Lagos State | Muritala Mohammed Way, Yaba | Moses A. Ojo | 8033652478 | ojoabayomimoses@yahoo.co.uk | Paid |
| The Wellness Center | 1A, Mojisola Onikoyi Street, Parkview Estate, Ikoyi, Lagos. | | | | Paid |
| Lagos State Drug Control Agency (LDSCA) | 36, Agege Motor Road, Mushin, Lagos. | | 807 704 7637 | | Paid |
| New Horizons Rehabilitation Center | 3, Olatunde Ayoola Crescent, Off Bode Thomas Street, Surulere, Lagos. | | | | Paid |
| The Bridge Rehabilitation Centre | 39, Akin Ogunlewe Street, Victoria Island, Lagos. | | | | Paid |
| House of Refuge Addiction Rehabilitation Center | 1, Prince Abubakar Audu Way, Off Oladipo Diya Road, GRA ,Ikeja. | | 8087290000 | | Paid |
| Resurgence Mental Health Support Initiative | 5b, Tokunbo Omisore, lekki phase 1 | Honest Anaba | 8034894587 | drhonestanaba@gmail.com | Paid |

| | | | | | |
|---|---|---|---|---|---|
| Christ Against Drug Abuse Ministry(CADAM) | 1 CADAM Drive Araga Epe, Lagos | Pst. Tunde Pelemo | 8063723949 | info@cadam.org.ng | Free |
| Tranquil And Quest Behavioural Health Center | 15 Isaac Olokun Aluko St, Igbo-Effon, 234001, Lagos | | 7069283293 | | Paid |
| Olive Prime Psychological Services | 10b Adewale Kolawale Crescent, TF Kuboye Drive, Lekki, Lagos | | 9057000852 | www.theoliveprime.com | Paid |
| Grace Hill Behavioural Health Services | 1 Patience Olukayode Crescent, Lakeview Estate, Off Raji Rasaki Estate Road, Amuwo Odofin, Lagos | Chigozirim Ebebi | 9091107514 | info@gmh.com.ng | Paid |
| HEDA Specialist Hospital | 2 Nwabueze Close Off Princess Aina Jegede Close Behind Eleganza Shopping Mall Ajao Estate Isolo, Lagos | Dr. Emmanuel Ohaka | 8033270656 | hedahospital@yahoo.com | Paid |
| A&D Referral Services, Surulere, Lagos. | 84 Ogunlana Street, Surulere, Lagos | | 9083574768 | | Paid |
| Wellspring Rehabilitation Centre | No.3, Banigo Close, off Akinrinmisi Street, off Agbedina Avenue, Isheri-Olowora, Lagos, | Buki Jinadu | 8070790121 | wellspring_ngo@yahoo.co.uk | Paid |
| Drug Addiction Rehabilitation and Mental Health | Mafoluku,Oshodi. | | 8039760351 | | Paid |

| | | | | | |
|---|---|---|---|---|---|
| NDLEA, Lagos Strategic Command, | 2Jogunomi street, Gbagada Phase II Estate Lagos. | | 8039760351, 8033478681 | | Paid (Subsidized) |
| Treasure Life Rehabilitation Centre | LASU, Igando Rd Lagos. | | 8057896733 | | Paid |
| SMART Recovery | 5b, Tokunbo Omisore, lekki phase 1 | Chinyere Okoro | 7037380350 | chinyerenduu @gmail.com | Free |
| ADICARE Rehabilitation Home | Ile epo ,Lagos. | | 8033319334 | | Paid |
| Serenity and Purpose Behavioural Center. | No 5 Olaseni-Diyaolu street off Ikenne Kilo Surulere Lagos Nigeria | | 7053018884 | | Paid |
