# Supplementary material for: Screening, brief intervention, and referral to treatment training for Nigerian primary care physicians: A pilot evaluation of knowledge, attitudes, self-efficacy, and barriers to implementation
Source: PLOS Glob Public Health. 2025 Dec 19;5(12):e0005597. doi: 10.1371/journal.pgph.0005597 (PMC12716713; doi:10.1371/journal.pgph.0005597)

JOHNS HOPKINS
BLOOMBERG SCHOOL
of PUBLIC HEALTH

LAGOS STATE
PRIMARY HEALTHCARE
BOARD

SBIRT Training for Lagos State Primary Care Physicians

Screening

Brief Intervention

Referral to Treatment

January 14–15, 2025 • 9AM

LCCI Conference and Exhibition Centre,
10 Dr Nurudeen Olowopopo Way, Lagos

SUPPORTED BY

Content Synopsis

- Overview of the SBIRT Approach
- The 5 As Steps
- Introduction to MI approach & process
- OARS skills
- Video demonstration of SBIRT using the MI approach
- Brief Intervention
- Brief Negotiated Intervention (BNI)
- Role Play

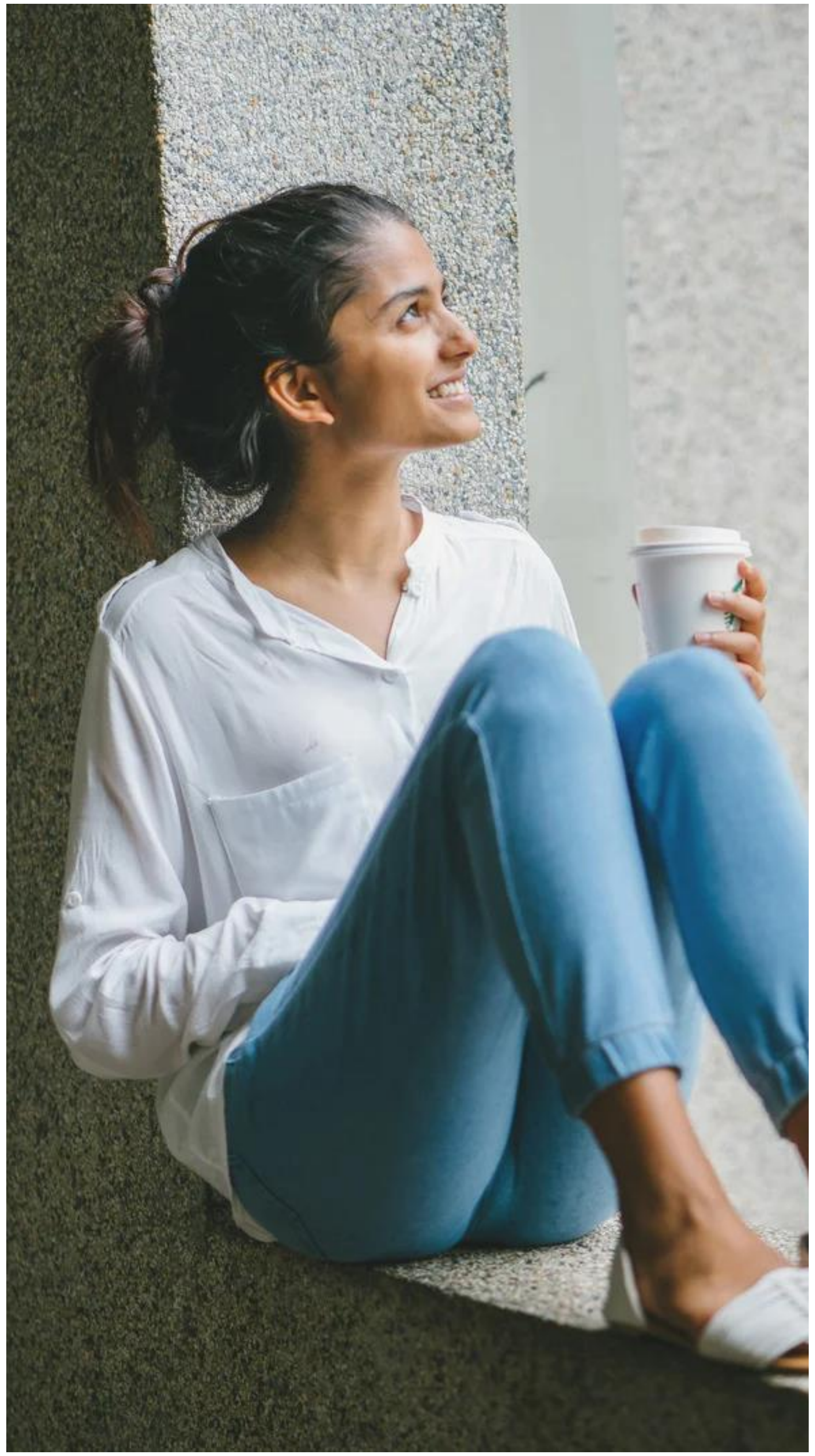

Overview of the SBIRT Approach

Presenter: **Chinyere Nduu Okoro (ICAP 2- Tx)**

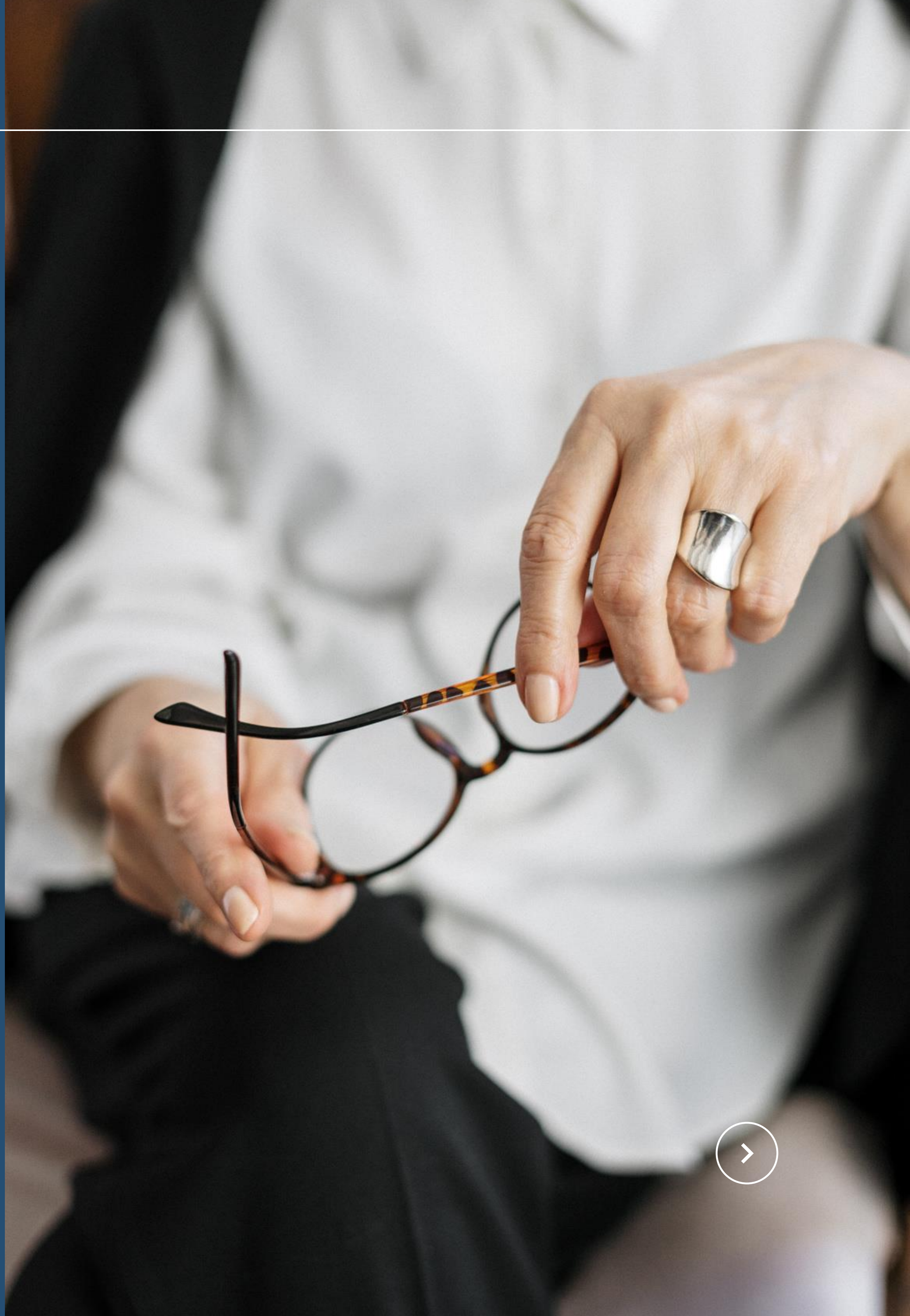

Screening, Brief Intervention and Referral to Treatment

AT THE INTERSECTION OF PREVENTION AND TREATMENT...

What is SBIRT?

SBIRT is a comprehensive**, integrated** public health approach to the delivery of **early intervention** and treatment services for persons with or at risk of substance use disorders.

SBIRT prevents further risks (SUD, accident, homelessness, etc) and opens a channel for those who need treatment to get it FAST.

People at higher risk include:

- Youths
- Those with mental health disorders
- People experiencing stressful and isolating times
- Those without family involvement
- People with chronic physical health ailments, etc.

Screening, Brief Intervention and Referral to Treatment

AT THE INTERSECTION OF PREVENTION AND TREATMENT...

Why SBIRT?

- Unhealthy alcohol and drug use are the most common causes of preventable morbidity and mortality.
- Unhealthy AOD (alcohol and other drugs) use often goes unrecognized in primary care.
- Research shows that a large number of people whose pattern of use puts them at high risk of developing substance use disorder (SUD) can be identified through screening.
- SBIRT is brief and is universally relevant and applicable.
- SBIRT decreases emergency department and hospital visits.
- SBIRT shows net-cost saving.

Screening, Brief Intervention and Referral to Treatment

Screening

Screening simply determines **whether a problem exists** or whether further assessment is needed. Screening should be conducted using a **validated brief instrument** to classify a patient's pattern of alcohol or drug use. **75-85%** of patients will screen negative. For those who screen positive, **further assessment** is needed to determine the level of risk.

Brief Intervention

Brief intervention (BI) is appropriate for patients identified through screening to be at moderate risk for substance use problems. BI **provides feedback** about unhealthy substance use. It also focuses on **education, increasing patient insight** and awareness about risks related to unhealthy substance use, and **enhancing motivation** toward healthy behavioural change. BI can be provided through a single session or multiple sessions of motivational interventions.

Screening, Brief Intervention and Referral to Treatment

Referral to Treatment

Patients identified as needing brief treatment or more intensive treatment than BI are **referred** to specialist SUD treatment providers. Referral to Treatment requires **coordination** with specialty care settings to ensure the referred patient can access and engage in appropriate level of care.

Continuum of Substance Use

What continuum exists?

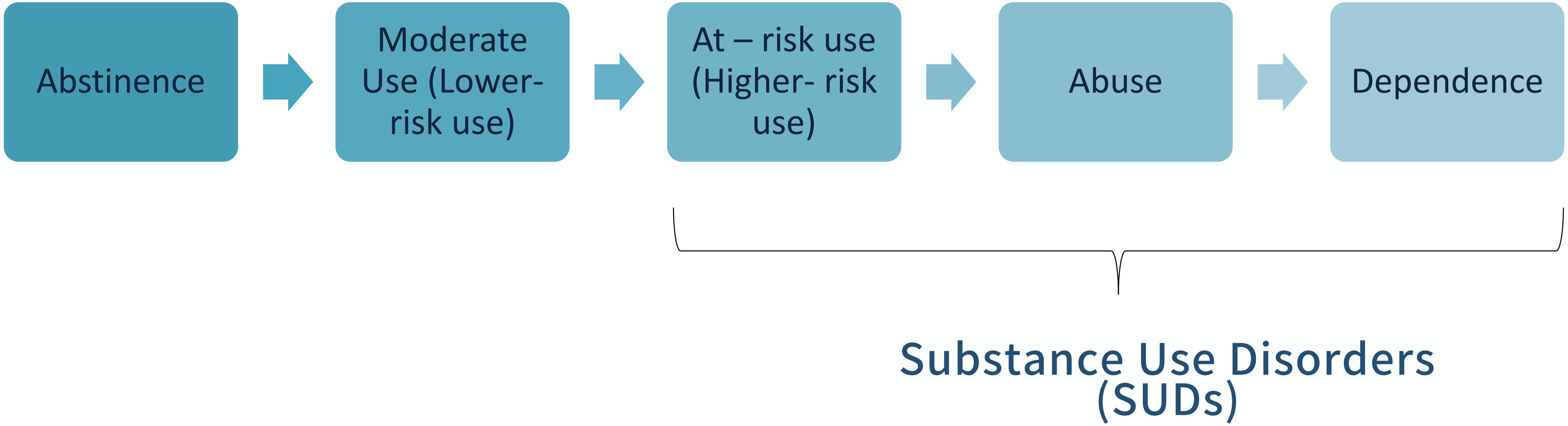

5 As: SBIRT Process

Referral to Treatment

- **Ask:** Establish rapport and enquire about substance use. Administer screening questions

"Hello Mr. /Mrs. /Ms…My name is ……We are now talking to each patient, no matter why they are here, about their alcohol and drug use…Is it all right if I ask you some questions?"

- **Advice:** Since most people will screen negative, offer commendation (don't praise) regarding patient's choices towards alcohol and drug use. For everyone, after screening, elicit feedback and offer psychoeducation about the impact of alcohol and drug use to mental and physical wellbeing.

"Your scores indicate that you are (summarize the results). What are your thoughts about this?... Would it be ok with you if I shared some information with you about how (specific substance use) can affect your …"

- **Assess**: Administer further questions to assess clients use for risk level.
- **Assist:** Assist patients with moderate and high-risk use develop a plan to cut-down use or abstain.
- **Arrange:** Arrange for referral to specialist treatment care for whom it is indicated.

SBIRT Method

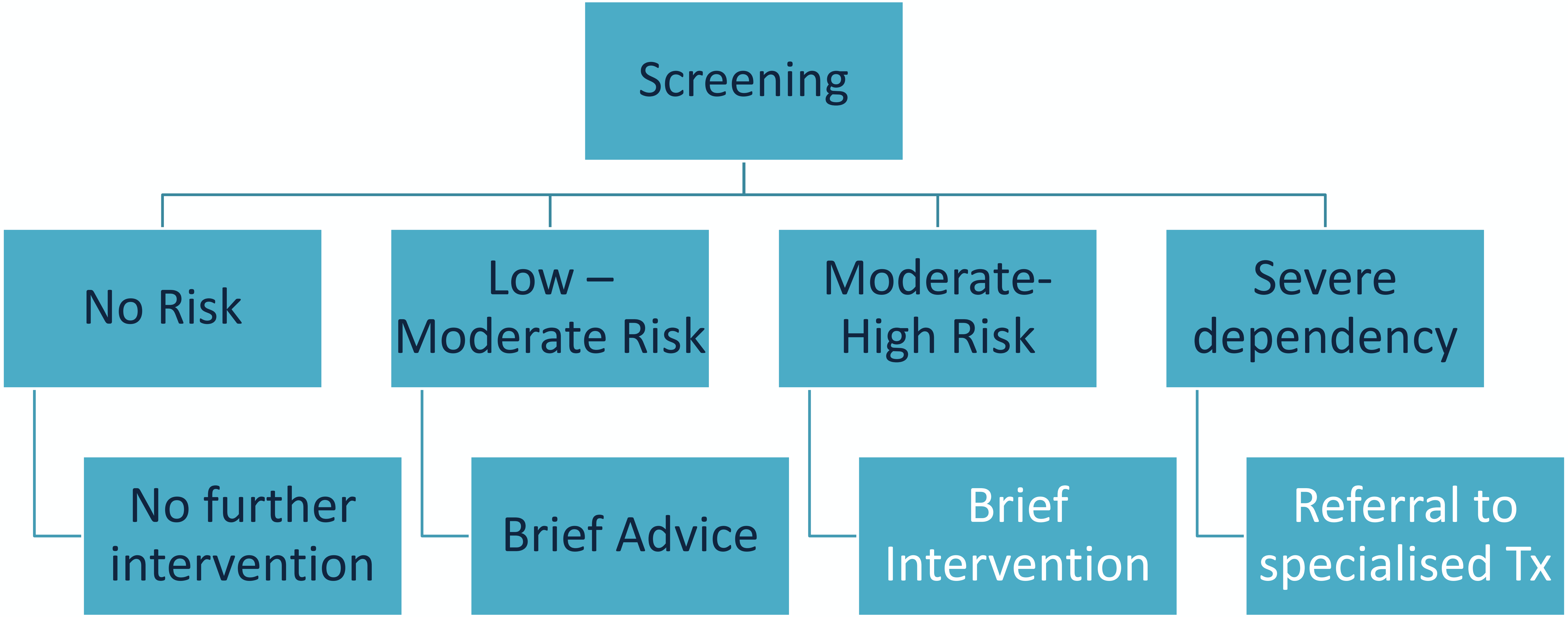

Age: 38

Occupation: Restaurant Manager

Background: Mark is a hard-working restaurant manager in Ajah who often juggles long hours, high stress, and demanding customers. He is married with two young children and describes himself as a social drinker and occasional smoker. However, his habits have changed over the past few years due to increased job pressures.

Mark visits your primary health clinic for a routine check-up. During the consultation, he casually mentioned feeling tired and irritable most of the time. When you ask about his lifestyle, **he admits to drinking alcohol "almost every evening"** to unwind after work and smoking a few cigarettes during his breaks. He also reveals he has **recently started using cannabis occasionally to "calm his nerves" on particularly stressful days.** Mark **smokes 5-10 cigarettes daily**, primarily during work breaks. He acknowledges that he's **been smoking for over a decade but says it's "manageable."** Even though **his wife has often told him that his smoking concerns her** and asked him to stop completely. Mark **drinks 2-3 beers daily** and report drinking a bit more during the weekends during outings. He doesn't think he has a drinking problem but admits he might drink more than that if situation calls for it. Additionally, Mark tell you a story about a time **8 months** ago, when he used his prescribed pain medication outside prescription just to calm down a bit. He said he did this twice.

Screen Mark using the TAPS and determine the risk level of his substance use behavior.

Motivational Interviewing

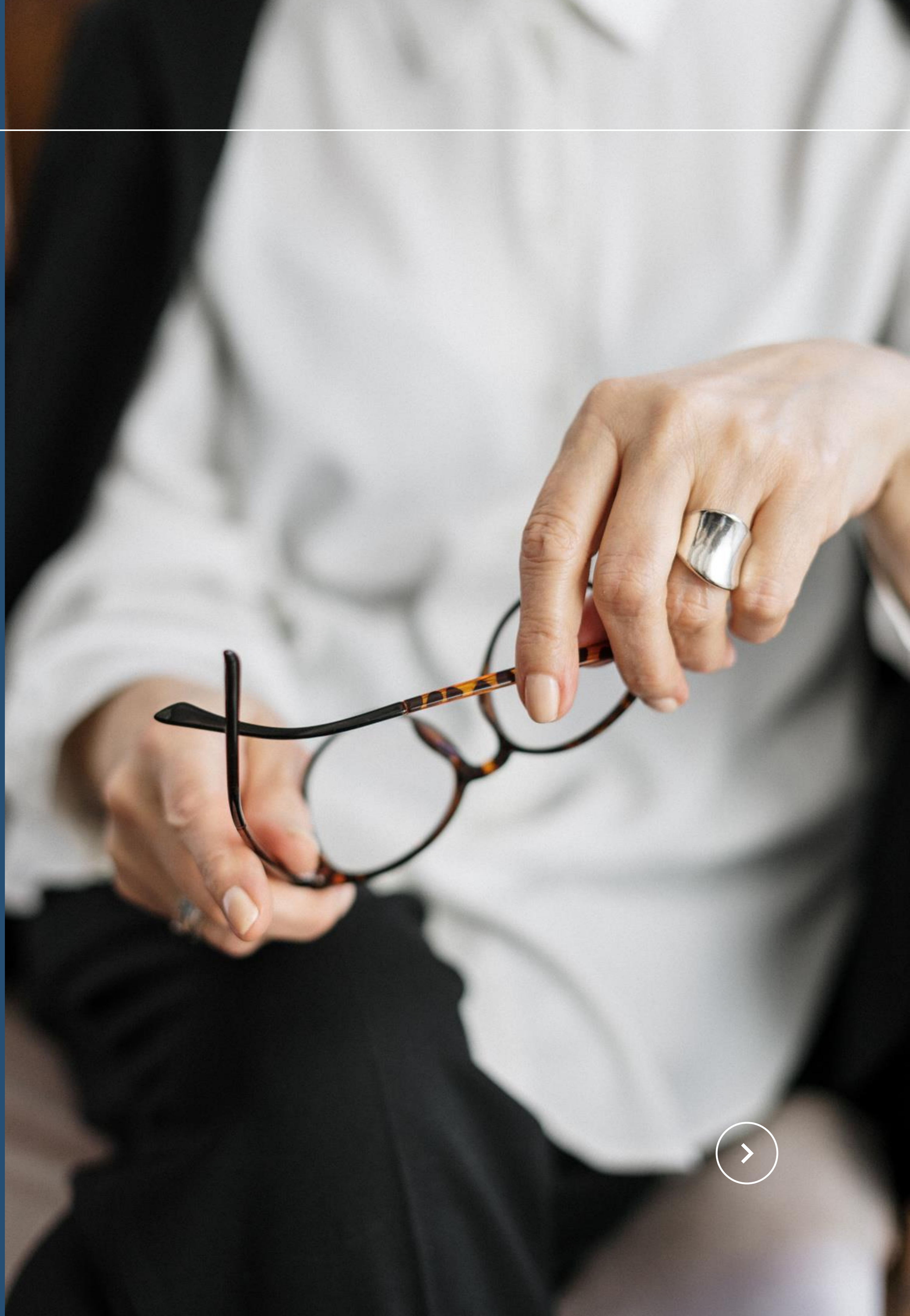

Presenter: **Chinyere Nduu Okoro (ICAP 2- Tx)**

How do you achieve this?

- Your patient to quit smoking
- Your teenage child to change friends
- Your toddler to eat something other than ice cream
- Your patient to take a vaccine

What is Motivational Interviewing?

MI:

Motivational Interviewing is both a treatment philosophy and a set of methods employed to help people increase internal motivation by exploring and resolving ambivalence about behavioral change. It is a directive **client-centered** style of interaction aimed at helping people to move through the stages of change.

Motivational Interviewing was developed by Willian R. Miller and Stephen Rollnick in the 1980s.

What is Motivational Interviewing?

Motivational Interviewing:

- Works
- Based on empathy which is associated with improved treatment outcome
- Builds efficacy
- Allows patient to own their decisions and plans for behavioural change
- Increases patient’s engagement in treatment
- Collaborative conversational style
- Goal-oriented
- Uses the language of change
- Strengthens personal motivation to change
- Elicits the person’s own reasons for change atmosphere of acceptance and compassion

Stages of Change

Precontemplation

- Problem? What problem! (Business as usual, not an SOC)

Contemplation

- Is this a problem? Do I want to change? Can I change?

Preparation

- How do I make this change? What am I going to do?

Action

- What works for me? What doesn't?

Maintenance

- How do I keep this change no matter what happens?

Skills of MI: OARS

Open-Ended Questions

- Encourage clients to do most of the talking, while the clinician **listens** and responds with a **reflection** or summary statement. The goal is to promote further dialogue that can be reflected back to the client by the clinician. Open-ended questions allow clients to tell their stories
- Open-ended questions are questions which require a longer answer and open the door for the person to talk.
- Examples of open ended questions include:

What are the good things about your substance use?

Tell me about the not so good things about using drug.

You seem to have some concerns about your substance use, tell me more about them.

What concerns you about…?

Skills of MI: OARS

Affirmation

- Affirmation include statements of appreciation and understanding helps to create a more supportive atmosphere, and helps build rapport with the patient. Affirming the patient's strengths and efforts to change helps build confidence, while affirming self-motivating statements (or change talk) encourages readiness.
- Examples of Affirmation includes:

Thanks for coming today.

I appreciate that you are willing to talk to me about your substance use.

You are obviously a resourceful person to have coped with those difficulties.

I can see that you are a really strong person.

That's a good idea.

Skills of MI: OARS

Reflective Listening

- Reflective listening response is a statement guessing at what the patient means. It is important to **reflect back** the underlying meanings and feelings the patient has expressed as well as the words they have used using reflective listening is like being a mirror for the person so that they can hear the physician say what they have communicated.
- In motivational interviewing reflective listening is used actively to highlight the patients ambivalence about their substance use, to steer the patient towards a greater recognition of their problems and concerns and to reinforce statements indicating that the patient is thinking about change.
- Examples of reflective statements includes:

You feel uncomfortable talking about this.

You're angry because your wife keeps nagging you about your substance use.

You are surprised that your score shows you are at risk of problems.

Skills of MI: OARS

Summary

- Summarizing is an important way of gathering together what has already been said and preparing the patient to move on summarizing adds to the power of reflective listening especially in relation to concerns and change talk. First patients hear themselves say it, then they hear the physician reflect it, and then they hear it again in. In summary, the therapist chooses what to include in the summary and can use it to change direction by emphasizing some things and not others. it is important to keep the summary succinct.
- **Practice**: Try summarizing the following:

“Yes, I know that I should have stayed with the children instead of going over to my friend’s house to party. But it wasn’t for such a long time. I don’t remember drinking that much. And anyway, that was nothing compared to getting locked out of my own house. When my wife got home she was so angry the kids were alone and I was at my friends. I don’t know what she’s going to do next. At least my friend was there for me and let me stay with him. He was a life-saver.”

Principles of MI: OARS

Express Empathy

- Accepting, non-judgmental approach that seeks to understand.

Develop Discrepancy

- Help patient see the difference between what they want and what they are doing.

Roll with resistance

- When the patient expresses resistance, do not argue, reframe it or reflect it rather than opposing it.

Support Self-efficacy

- The physician's belief in the patient's ability to change their behavior is also important and can become a self-fulfilling prophecy.

Video Demonstration

https://www.youtube.com/watch?v=67I6g1I7Zao

https://www.youtube.com/@SBIRTOregon : Brief Intervention Videos

Brief Intervention

Presenter: **Chinyere Nduu Okoro (ICAP 2- Tx)**

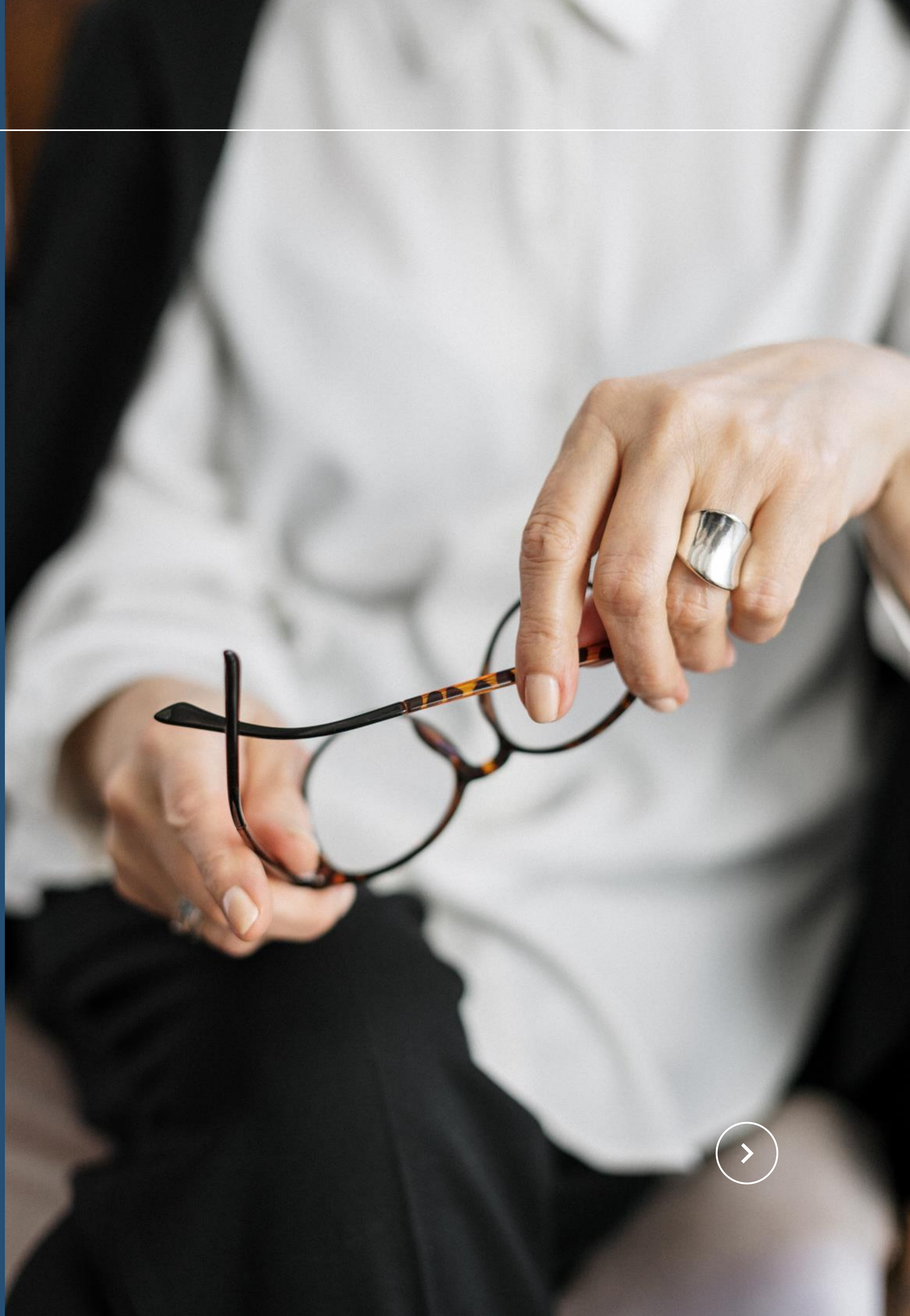

What is Brief Intervention?

Brief Intervention

Brief Intervention is a **brief motivational** and **awareness-raising** intervention given to risky or problematic substance users.

There are several models for brief intervention. There is motivational interviewing, FRAMES, WHO ASSIST-linked 10-step BI, The 5 As, Making Every Contact Count (MECC), etc

The Brief Negotiated Intervention (BNI) is a semi-structured interview process based on MI that is a proven evidence-based practice and can be completed in 5−15 minutes.

It is developed by Gail D'Onofrio, M.D., Ed Bernstein, M.D., Judith Bernstein, M.S.N., Ph.D., and Steven Rollnick, Ph.D.

Steps in Brief Negotiated Intervention (BNI)

How do we get it done?

- Build rapport - Raise the subject
- Provide feedback
- Build readiness to change
- Negotiate a plan to change

Step 1: Build Rapport and Raise the Subject

1. **Begin with a general conversation.**
“Hello Mr, Mrs, Ms, ….. How are you doing today? We are now speaking to every patient about their alcohol and drug use…”

2. **Ask permission to talk about alcohol and drug use.**
“…is it okay if I ask you some questions? Here is a simple and brief screening questionnaire we can fill out together, are you comfortable to participate?…”

3. **Assure of confidentiality**
“Everything you tell me is completely confidential and will be shared only with your health care providers here at this clinic. Do you have any questions or concerns?”

Step 1: Build Rapport and Raise the Subject

What if the patient does not want to talk about their use?

Step 1: Build Rapport and Raise the Subject, Discuss Pro and Cons

"Help me understand, through your eyes:

- What are the **good things** about smoking cigarette?

- What are the **not so good things** about smoking cigarette?

Step 1: Build Rapport and Raise the Subject, Discuss Pro and Cons (Apply MI)

Use Open-ended questions

Enable patient to convey more information
Encourage engagement
Opens the doors for exploration and understanding

Use Reflections

Reflective listening
Thinking reflectively

Summarizing

Reinforce what has been said
Shows careful **listening**

Step 2: Provide Feedback

- Ask permission to give information
- Discuss screening findings
- Link substance use behavior to any known consequences (already share in step 1 above)
- Evoke a response

"What do you think about these scores...? How does hearing this feedback make you feel?..."

- Positive response? *("…I never thought my alcohol drinking could be affecting me this much. I would like to change things…")* **– Move forward with Brief Intervention**
- Negative response? *("…I do not see any problem with my use still…")* – **Revisit the pros and cons**

Step 3: Build Readiness to Change

"Can we talk for a few minutes about your interest in making a change?

(Get consent and move on)

On a scale from 1-10 with 1 being not ready at all and 10 being completely ready, how ready are you to make any changes in your substance use?"

1	2	3	4	5	6	7	8	9	10
Not at all ready				Somewhat ready					Extremely ready

Step 3: Build Readiness to Change

Explore client motivation and barriers:

"Why that number and not XX (lower number)? (Change talk)
Why that number and not XX (higher number)? (Barriers for change)"

Explore patient's idea about implementing change
"What steps if any, might you take from here?"

Step 4: Negotiate a Change Plan

A plan to reduce use to low-risk levels

OR

An agreement to follow-up with specialty treatment care

"Can you think of one thing you could do to make a change in the next month?
Never mind the "how" for right now. What would you like to achieve?
So what do you intend to do?
What strengths do you have that will help you succeed?
Who could offer you helpful support in making this change?"

Video Demonstration

Video Demonstration:
https://www.youtube.com/watch?v=g2v2sfwfQ84

https://www.youtube.com/watch?v=7nevSz5-dhc

https://www.youtube.com/watch?v=F5fMkat8aJE

https://www.youtube.com/watch?v=MaxHuf17A44

Role play 1

Meet Mr. John

John, a 45-year-old male construction worker, visited his local primary health clinic where you work, complaining of a persistent cough and shortness of breath, especially during physical activity. During the consultation, you conducted a routine assessment and noted that John has been smoking 20 cigarettes a day for over 20 years. Mr. Johns TAPS reported a score 4 out of 4. He reported frequent cravings for cigarettes, particularly during work breaks, and admitted he hadn't seriously considered quitting.

How would you administer Brief Negotiated Intervention for Mr. John?

Role play

<u>Roles:</u>

Patient

Interviewer

Observer

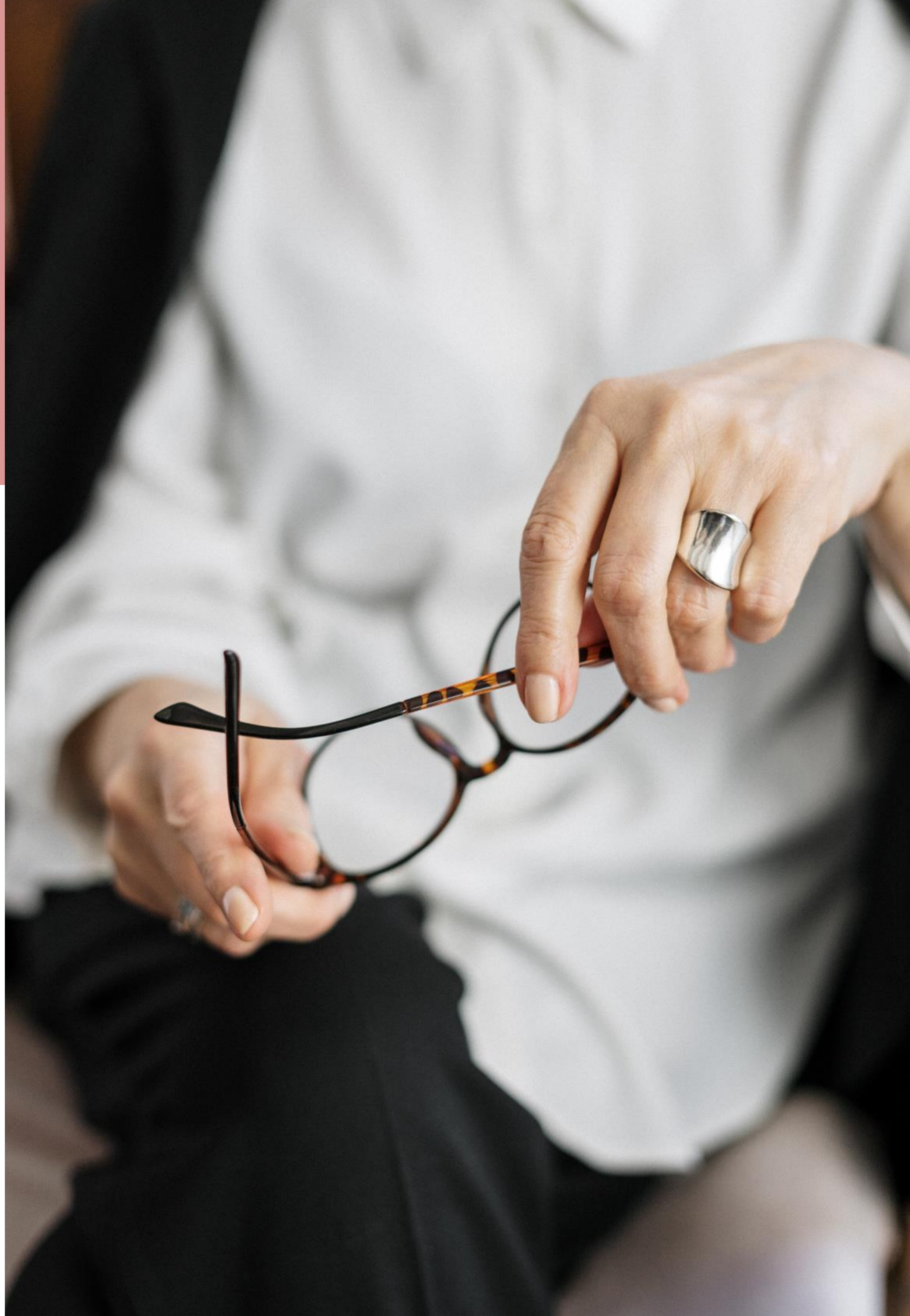

Role play : Feedback

What went well?

What did not go so well?

What do you think could have been done differently?

Closing Reflections:

Based on your performance in all the practice scenarios, what skills do you think you perform well? Which of your skills needs practice so you can more effectively help your patients?

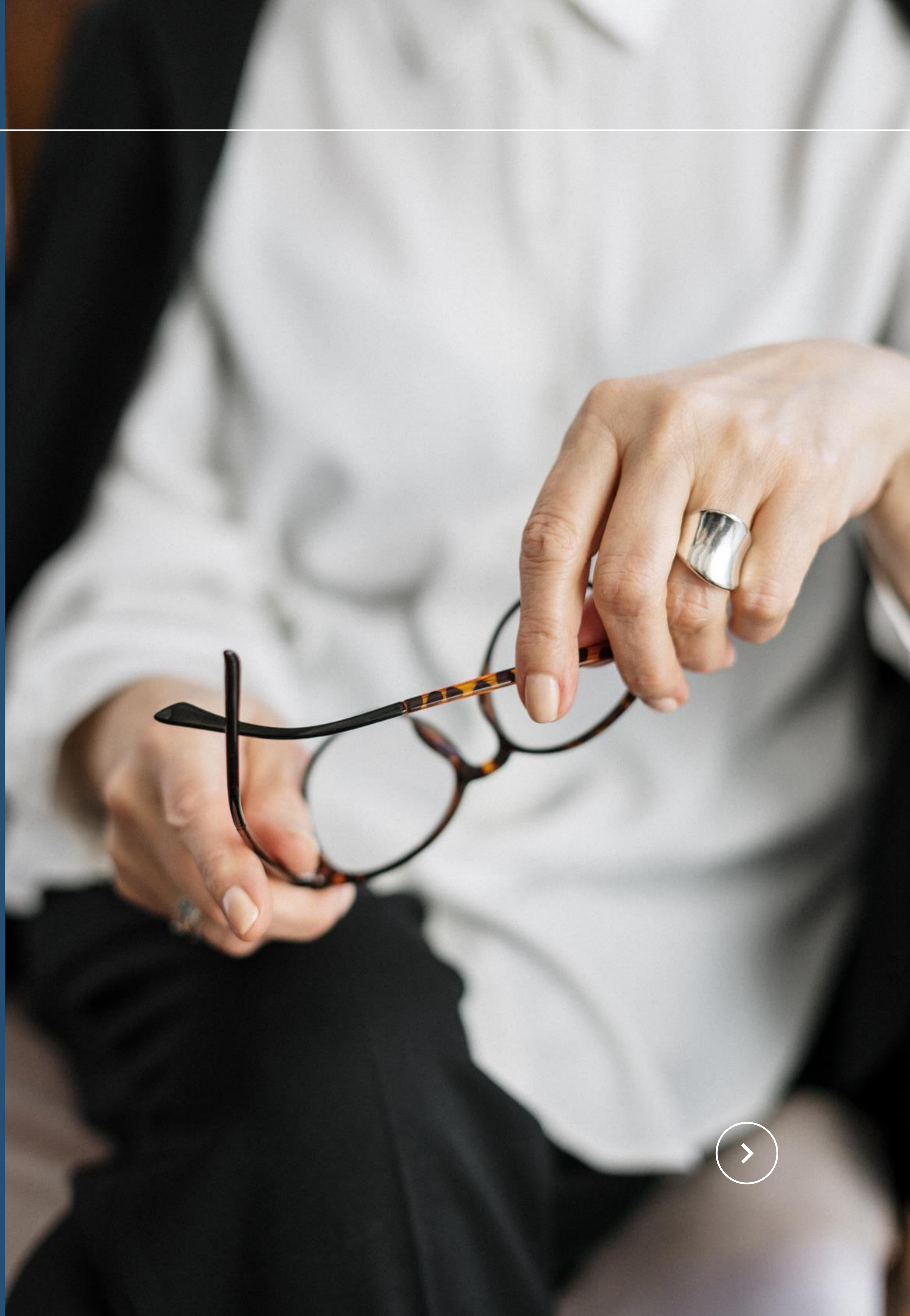

References:

1. SAMHSA Brief Negotiated Intervention Training
2. Technical Assistance Publication Series (TAP 33) System-level Implementation of Screening, Brief Intervention and Referral to Treatment, Substance Abuse and Mental Health Services Administration (SAMHSA)
3. International Society of Substance Use Professional (ISSUP) Universal Treatment Curriculum- 11 (Motivational Interviewing)
4. International Society of Substance Use Professional (ISSUP) Webinar Slides, SBIRT for Substance Use, Shaheema Allie Fergus Ashburner Nurain Tisaker, November 2023.
5. Bureau of Substance Use Services, SBIRT Step by Step guide, Clinician guide
6. WHO ASSIST-linked Brief Intervention Guide
7. SBIRT Step-by-Step

Questions?

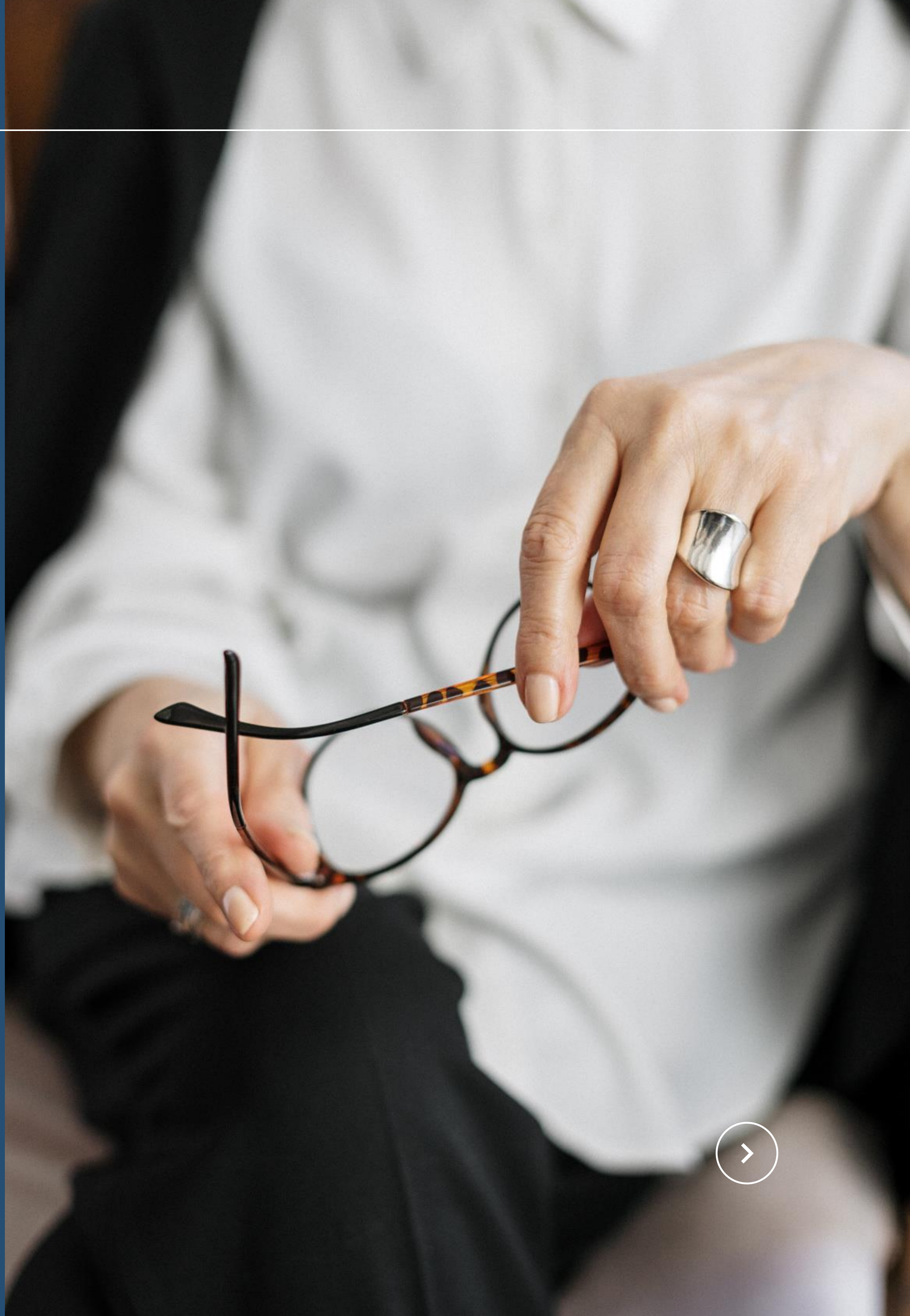

Supplement: S3 File — (PDF) [file pgph.0005597.s003.pdf]
