# Supplementary material for: Screening, brief intervention, and referral to treatment training for Nigerian primary care physicians: A pilot evaluation of knowledge, attitudes, self-efficacy, and barriers to implementation
Source: PLOS Glob Public Health. 2025 Dec 19;5(12):e0005597. doi: 10.1371/journal.pgph.0005597 (PMC12716713; doi:10.1371/journal.pgph.0005597)
Supplement: S4 File — (DOCX) [file pgph.0005597.s004.docx]

**Pre - Survey questionnaire: knowledge, perception, attitudes, self-efficacy**

**Survey Questions:**

Please know any information provided will be kept strictly confidential

**Socio-demographic questions:**

1. What is your age? (18–24, 25–34, 35–44, 45–54, 55+)
2. What is your gender? (Male/Female/Prefer not to say)
3. What is your current marital status? (Single/Married/Divorced/Widowed/Other)
4. How many years have you worked in healthcare? (Less than 5 years, 6–10 years, 11+ years)
5. What is your religious affiliation? (Christianity/Islam/Traditional/Other/None)
6. How often do you encounter patients with substance use issues in your practice? (Not sure/Never/Rarely/Occasionally/Frequently)
7. Have you received any formal training on screening for substance use? (Yes/No/Not sure)

1**. Knowledge:**

1. Are you familiar with screening tools used to assess drug use (e.g., ASSIST, AUDIT, TAPS)? (Yes/No/)
2. Do you know the key signs and symptoms of substance abuse? (Yes/No)
3. How would you rate your level of knowledge in screening patients for drug use? (Poor/Average/Good/Excellent)
4. Do you know of any guidelines for assessing drug use in Nigeria? (Yes/No)
5. Have you heard of SBIRT as a framework for addressing substance in Nigeria? (Yes/No)

2. **Perception and Attitudes** of Healthcare Workers Regarding SBIRT (Screening, Brief Intervention, and Referral to Treatment) in Nigeria

1. Primary care providers can play a significant role in addressing substance use disorders (Yes/No/Not sure)
2. How effective do you think brief interventions are in addressing substance use issues in primary care? (Not Effective/Somewhat Effective/Effective/Very Effective)
3. Should all patients with substance use be referred to specialized treatment centres (Yes/No/Not sure)
4. How likely are you to treat patients with substance use issues with the same respect as other patients? (Not Likely/Somewhat Likely/Likely/Very Likely)
5. Substance use is a medical issue, not a moral failing (Strongly Agree/Agree/Neutral/Disagree/Strongly Disagree)

3. **Self-Efficacy** of Healthcare Workers in Assessing substance use in Nigeria

1. How confident are you in starting conversations about substance use with patients? (Not Confident/Somewhat Confident/Confident/Very Confident)
2. How confident are you in using screening tools (e.g., ASSIST, AUDIT, TAPS,etc)? (Not Confident/Somewhat Confident/Confident/Very Confident)
3. How confident are you in providing brief interventions? (Not Confident/Somewhat Confident/Confident/Very Confident)
4. How confident are you referring patients with substance use issues to appropriate care? (Not Confident/Somewhat Confident/Confident/Very Confident)
5. What factors most affect your ability to assess substance use? (Lack of Training/Time Constraints/Patient Resistance/Systemic Issues/Other)
6. Would additional training increase your confidence in assessing substance use cases? (Yes/No/Not sure)

**Post-survey questionnaire**

Participant details:

**Section 2: Training Feedback**

1. How satisfied are you with the training overall? (Very Dissatisfied/Dissatisfied/Neutral/Satisfied/Very Satisfied)
2. How clear and practical were the training materials? (Very Poor/Poor/Average/Good/Excellent)
3. How effective were the trainers in facilitating the sessions? (Very Poor/Poor/Average/Good/Excellent)
4. Were the training sessions interactive and engaging? (Strongly Disagree/Disagree/Neutral/Agree/Strongly Agree)
5. How satisfied were you with the duration of the training? (Very Dissatisfied/Dissatisfied/Neutral/Satisfied/Very Satisfied)

**Section 3:
Knowledge:**

1. Rate your knowledge in screening for drug use (Poor/Average/Good/Excellent)
2. Which skills did you improve the most during the training? (Select all that apply)

- Identifying substance use issues
- Communicating effectively with patients
- Conducting a brief intervention
- Making appropriate referrals
- Other: ___________________

**Perception and Attitudes:**

1. How likely are you to treat patients with substance use issues with the same respect as other patients with other chronic medical conditions? (Not Likely/Somewhat Likely/Likely/Very Likely)
2. The training content was relevant to your role. (Strongly Disagree/Disagree/Neutral/Agree/Strongly Agree)

**Self-Efficacy:**

1. How confident are you in starting conversations about screening in your daily practice? (Not Confident/Somewhat Confident/Confident/Very Confident)
2. Rate your confidence of the following SBIRT components after training:

- Screening for substance use:(Not Confident/Somewhat Confident/Confident/Very Confident)
- Brief intervention techniques: (Not Confident/Somewhat Confident/Confident/Very Confident)
- Referral to treatment: (Not Confident/Somewhat Confident/Confident/Very Confident)

1. Do you feel prepared to handle challenges in SBIRT implementation (Strongly Disagree/Disagree/Neutral/Agree/Strongly Agree)

**Section 4: Implementation and Future Support**

1. How likely are you to implement SBIRT in your practice under 3 months? (Not Likely/Somewhat Likely/Likely/Very Likely)
2. What barriers do you anticipate in implementing SBIRT? (Open-ended)
3. What additional resources or support would help you apply SBIRT effectively? (Open-ended)
4. Would you recommend this training to other healthcare workers? (Yes/No/Not Sure)
5. Please share any suggestions to improve this training: (Open-ended)
