# Supplementary figures and images for: Screening, brief intervention, and referral to treatment training for Nigerian primary care physicians: A pilot evaluation of knowledge, attitudes, self-efficacy, and barriers to implementation
Source: PLOS Glob Public Health. 2025 Dec 19;5(12):e0005597. doi: 10.1371/journal.pgph.0005597 (PMC12716713; doi:10.1371/journal.pgph.0005597)

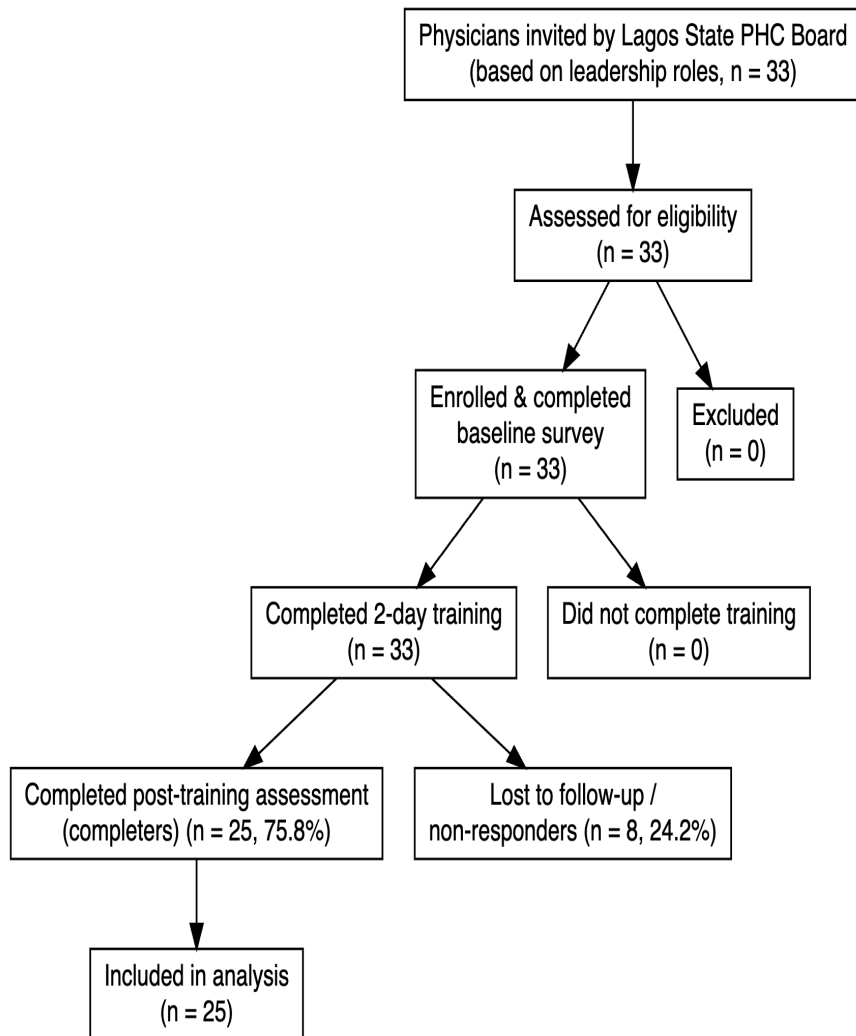

Supplement: S1 Fig — (PDF) [file pgph.0005597.s005.pdf]
