# Supplementary material for: Screening, brief intervention, and referral to treatment training for Nigerian primary care physicians: A pilot evaluation of knowledge, attitudes, self-efficacy, and barriers to implementation
Source: PLOS Glob Public Health. 2025 Dec 19;5(12):e0005597. doi: 10.1371/journal.pgph.0005597 (PMC12716713; doi:10.1371/journal.pgph.0005597)
Supplement: S5 File — (PDF) [file pgph.0005597.s006.pdf]

TIDieR Checklist – SBIRT Training for Nigerian Primary Care Physicians

This checklist corresponds to the manuscript titled "Screening, Brief Intervention, and Referral to Treatment (SBIRT) Training for Nigerian Primary Care Physicians: A Pilot Evaluation of Knowledge, Attitudes, Self-Efficacy, and Barriers to Implementation.

The page/section column identifies where each intervention element is reported within the manuscript.

TIDieR Item	Brief Description	Page / Section in Manuscript
**1. Brief name**	"Screening, Brief Intervention, and Referral to Treatment (SBIRT) training."	Title & Abstract, p. 1
**2. Why**	Address knowledge gaps in SBIRT, reduce stigma toward SUD, and build delivery confidence among PHC physicians to support Nigeria's National Drug Control Master Plan (2021-2025) integration goals	Introduction, p. 2–4
**3. What – materials**	Training slide deck, video demonstrations, screening tool handouts (ASSIST, TAPS, AUDIT), Brief Negotiated Interview (BNI) scripts, Lagos substance use treatment and recovery resource directory, information leaflets and CME certificates. Full curriculum provided in S1 Appendix	Methods – Intervention, p. 5–6; S2 Appendix
**4. What – procedures**	Two-day workshop content (epidemiology, screening tools TAPS/ASSIST, MI skills, BNI, stigma, role-play).	Methods – Intervention procedures, p. 5–6
**5. Who provided**	Lead trainers:<br>Dr. Honest Anaba (Addiction physician), Chinyere Okoro (Addiction counselor and SMART Master Trainer),<br>Angela Bekederemo (Clinical social worker and Addiction Specialist).<br>Guest speakers: Lagos state Primary healthcare board acting permanent secretary, Lagos State NDLEA Commander, NAFDAC Director, NMA Lagos Chair, ISSUP Nigeria Lagos Chair.<br>Teaching assistants: Olusesan Kayode (UNODC Master trainer), Dr. Elohor Oborevwori (Public health physician), and peer trainers with SBIRT experience.	Methods – Intervention & Acknowledgements, p. 17
**6. How (delivery mode)**	In-person workshop; instructional methods: didactic presentations, video demonstrations, small-group discussions, interactive role-play exercises (brief intervention practice), incentive-based Q&A, daily evaluations.	Methods – Intervention delivery mode, p. 6

**7. Where**	Two consecutive days (14-15 January 2025), 8:30am–3:30pm (approximately 13 hours total instructional time over 2 days, excluding breaks/lunch)	Methods – Study Design & Population, p. 5; Figure 1
**8. When and how much**	Two-day workshop (Jan 14–15, 2025); single train-the-trainer course; 10 CME credits.	Methods – Intervention, p. 5–6
**9. Tailoring**	Content adapted for Nigerian context: local substance use epidemiology ("Drug Situation in Nigeria" module), culturally relevant case examples, referral pathways mapped to Lagos-specific treatment resources, stigma module addressing local cultural attitudes toward SUD	Methods – Intervention, p. 5–6
**10. Modifications**	None reported during study.	N/A – No modifications described
**11. How well – planned fidelity**	No formal fidelity assessment conducted. Trainers followed structured agenda; participant engagement monitored through daily evaluations.<br>Limitation: Future iterations should include fidelity checklists, independent session observations, and standardized delivery protocols.	Methods – Intervention, p. 5–6
**12. How well – actual fidelity**	Not formally assessed. All sessions completed; high participant satisfaction noted, but protocol adherence and delivery quality not independently verified.	Results – Training Satisfaction, p. 10–11
